# Transient Peripheral Immune Activation follows Elective Sigmoidoscopy or Circumcision in a Cohort Study of MSM at Risk of HIV Infection

**DOI:** 10.1371/journal.pone.0160487

**Published:** 2016-08-18

**Authors:** Javier R. Lama, Shelly T. Karuna, Shannon P. Grant, Edith M. Swann, Carmela Ganoza, Patricia Segura, Silvia M. Montano, Martin Lacherre, Stephen C. De Rosa, Susan Buchbinder, Jorge Sanchez, M. Juliana McElrath, Maria P. Lemos, HVTN 914 Study Team

**Affiliations:** 1 Asociacion Civil Impacta Salud y Educacion, Lima, Peru; 2 Vaccine and Infectious Disease Division, Fred Hutchinson Cancer Research Center, Seattle, Washington, United States of America; 3 Vaccine Clinical Research Branch, Division of AIDS, National Institutes of Allergy and Infectious Diseases, US National Institutes of Health, Bethesda, Maryland, United States of America; 4 US Naval Medical Research Unit No. 6, Callao, Peru; 5 Department of Global Health, University of Washington, Seattle, Washington, United States of America; 6 Department of Laboratory Medicine, University of Washington, Seattle, Washington, United States of America; 7 Department of Medicine, University of Washington, Seattle, Washington, United States of America; 8 San Francisco Department of Health, San Francisco, California, United States of America; Rush University, UNITED STATES

## Abstract

**Background:**

Rectal and genital sampling in HIV prevention trials permits assessments at the site of HIV entry. Yet the safety and acceptability of circumcision and sigmoidoscopy (and associated abstinence recommendations) are unknown in uncircumcised men who have sex with men (MSM) at high risk of HIV infection.

**Methods:**

Twenty-nine HIV-seronegative high-risk Peruvian MSM agreed to elective sigmoidoscopy biopsy collections (weeks 2 and 27) and circumcision (week 4) in a 28-week cohort study designed to mimic an HIV vaccine study mucosal collection protocol. We monitored adherence to abstinence recommendations, procedure-related complications, HIV infections, peripheral immune activation, and retention.

**Results:**

Twenty-three (79.3%) underwent a first sigmoidoscopy, 21 (72.4%) were circumcised, and 16 (55.2%) completed a second sigmoidoscopy during the study period. All who underwent procedures completed the associated follow-up safety visits. Those completing the procedures reported they were well tolerated, and complication rates were similar to those reported in the literature. Immune activation was detected during the healing period (1 week post-sigmoidoscopy, 6 weeks post-circumcision), including increases in CCR5^+^CD4^+^T cells and α4β7^+^CD4^+^T cells. Most participants adhered to post-circumcision abstinence recommendations whereas reduced adherence occurred post-sigmoidoscopy.

**Conclusion:**

Rectosigmoid mucosal and genital tissue collections were safe in high-risk MSM. Although the clinical implications of the post-procedure increase in peripheral immune activation markers are unknown, they reinforce the need to provide ongoing risk reduction counseling and support for post-procedure abstinence recommendations. Future HIV vaccine studies should also consider the effects of mucosal and tissue collections on peripheral blood endpoints in trial design and analysis.

**Trial Registration:**

ClinicalTrials.gov NCT02630082

## Introduction

HIV exposure in rectal and genital compartments varies dramatically in HIV transmission risk, with receptive anal intercourse (RAI) being 12–35 times more likely to lead to infection that penile insertive behaviors [1, 2]. Such differences in compartments may require distinct immune potency and/or different mechanisms of protection to prevent HIV infection. In addition, rectal and genital sites can exhibit a degree of immune compartmentalization that cannot be studied from blood sampling [3–7]. Thus, to fill gaps in our understanding of immune responses to HIV vaccines, studies of mucosal immunity aim to complement assessments of systemic immunity. However, it is unclear and important to understand if intestinal and genital sampling can impact peripheral blood endpoints in HIV vaccine trials.

The collection of secretions is the least invasive method of sampling mucosal compartments, but it is limited to assessments of secreted soluble mediators, such as cytokines and antibodies. Secretions are not representative of the conditions of HIV-exposed mucosal tissue, where cellular and humoral components of a vaccine can act to prevent cell-associated and free virus transmission [8–10]. Therefore, genital and intestinal tissue sampling might be needed to fully understand vaccine responses in humans. For example, in the Step Study the lack of information about mucosal tissue responses to the vaccine in Ad5 seropositive individuals has made it difficult to interpret the increased risk observed with vaccination, or its association with circumcision status [11]. T-cell mediated vaccines such as those using rhesus cytomegalovirus vectors may also benefit from tissue sampling at HIV exposure sites, where effector-memory T cells could play a significant role in the immune response [10].

In this manner, large efficacy trials in populations at risk of HIV have been complemented by smaller trials with mucosal sampling in populations at low HIV risk. However, multiple differences among mucosal compartments in high risk and low risk individuals, such as inflammation, the presence of other sexually transmitted infections (STIs), and potential microtrauma relating to sexual activity, can confound extrapolation of intestinal and genital response findings from low risk individuals to high risk ones [12–14]. Thus, some data from mucosal studies in low risk individuals may not represent mucosal and genital compartments of the populations at risk of HIV.

One gap in the HIV prevention clinical trials field is in our understanding of whether genital and intestinal sampling and the associated peri-procedure behavioral recommendations are tolerable, acceptable, and safe to sexually active men who have sex with men (MSM). For example, the acceptability and safety of circumcision was demonstrated in a largely heterosexual and HIV-negative population in East and South Africa [15–17], but data regarding circumcision in MSM is less clear [18]. Although East and South African circumcision trials reported high adherence to post-procedure sexual abstinence recommendations and low complication rates [15–17, 19, 20], levels of baseline sexual activity in those populations varied, and complication rates were higher with earlier post-procedure resumption of sexual activity. A better understanding of adherence to post-procedure abstinence in sexually active MSM in the Americas is needed.

Information about the acceptability and safety of sigmoidoscopy with biopsy in high-risk MSM is even more limited than that for circumcision. Few groups have conducted flexible sigmoidoscopy studies in participants who practice RAI [21, 22]. Therefore, to better characterize foreskin and mucosal immune responses and understand the acceptability and safety of circumcision and rectosigmoid biopsies in high-risk South American MSM, the HIV Vaccine Trials Network (HVTN) conducted HVTN 914, a cohort study among MSM at high risk for HIV infection in Lima, Peru.

## Methods

### Ethics Approvals

The study protocol and materials were approved by Institutional Bioethics Committee/Review Boards from Asociacion Civil Impacta Salud y Education (IMPACTA), US Naval Medical Research Unit No. 6, and the Fred Hutchinson Cancer Research Center. All study participants provided written informed consent prior to participation. Because the trial does not evaluate experimental interventions (circumcision and flexible sigmoidoscopy procedures are well established in clinical practice), the protocol team did not register the trial in ClinicalTrials.gov before participant enrollment. Upon request of the journal, the registration was completed (ClinicalTrials.gov NCT02630082). The study protocol and supporting TREND checklist are available as supplemental information; see S1 Protocol and S1 Checklist. The authors confirm that all ongoing and related trials for these interventions are registered.

### Study Population

In Lima, Peru, the MSM population has an HIV seroprevalence that is a 40-fold higher than the general male population [23], and high HIV risk MSM experience a 2.2–3.5% HIV incidence rate [11, 24]. HVTN 914 recruited healthy, HIV-seronegative, uncircumcised MSM 21 to 30 years old, who reported one or both criteria for HIV acquisition: 1) anal intercourse without condoms with one or more male or male to female transgender (MTF) partners in the preceding six months, and 2) reporting anal intercourse with two or more male or MTF partners in the preceding six months. Volunteers who had been in a monogamous relationship with an HIV-seronegative partner for > 6 months were excluded from participation due to low HIV transmission risk. Volunteers having sex with known HIV positive partners or a history of transactional sex in the past six months were ineligible due to concern for potential post-procedure exposures during healing, and/or economic difficulty adhering to abstinence recomendations.

Each participant’s healthy status was defined by medical history, physical examination, and having a normal hematology and coagulation profile. Men with a medical indication for circumcision (chronic balanitis or phimosis), foreskin covering less than half the glans, history of keloid scarring, or untreated clinical evidence of genitourinary or colonic infection were excluded.

### Recruitment and Screening Procedures

A combination of social media and outreach was used to recruit participants from April 2011 through October 2012. No recruitment activity promoted male circumcision for HIV prevention, given the lack of evidence for MSM [18].

At the screening visit, a counselor obtained informed consent for study participation at the clinic. It included willingness to receive risk reduction counseling and HIV testing, described risks and benefits of circumcision and of sigmoidoscopy with biopsies, and described peri-procedure safety protocols. Participants were also informed about who had access to their personal information and study data. A different staff member conducted an assessment of understanding and clarified any misconceptions of key concepts. Screening included coagulation assessments, HIV-1/2 and Herpes Simplex Virus 2 (HSV-2) serology, and physical examination.

### Enrollment and Follow-Up Study Visits

**Fig 1:**
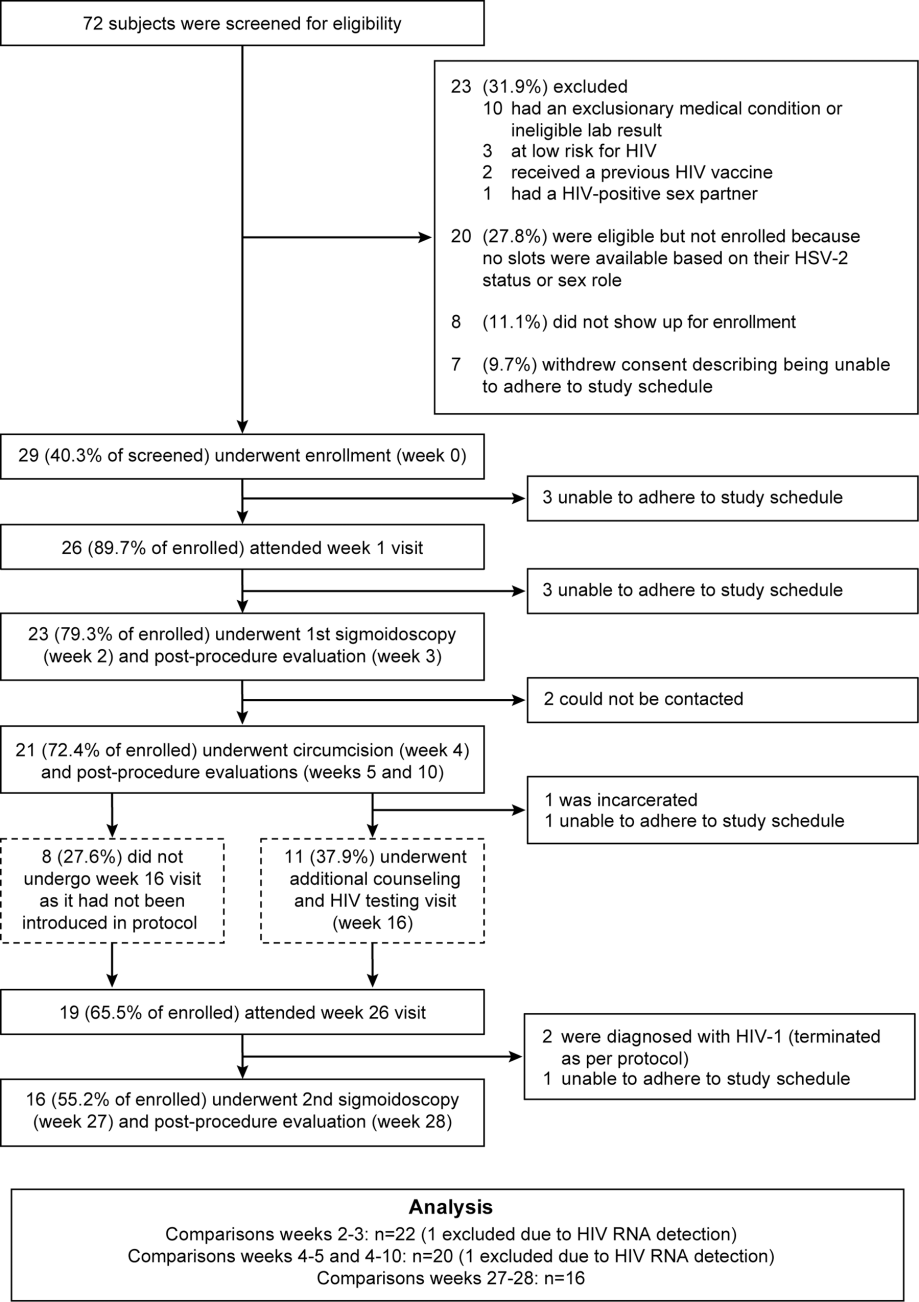
Study Conduct. Flow chart indicates the number and percentage (%) of participants at screening who underwent procedures at each study visit. Also included are those who left the study, and the reasons why. The dashed boxes indicate the visit added after 8 participants had already completed the protocol.

Fig 1 details screening, enrollment, and follow-up in Lima, Peru. The first 12 enrolled participants were scheduled to attend 10 visits during the 28 week study. They were tested for HIV-1/2 and HSV-2 at screening and at week 26. Six months into the protocol, another HIV test at week 16 was added for remaining participants to improve HIV surveillance. Syphilis was assessed at enrollment (week 0), *Neisseria gonorrhea*, and *Chlamydia trachomatis* were assessed at weeks 0 and 26. Participants diagnosed with any STI received treatment following standard guidelines [25].

To ensure representation of MSM with different sexual behaviors [1] and differential HIV acquisition risk [26–28], we recruited a maximum of 5 men into each of 6 groups defined by self-reported anal sex preferences in preceding six months (insertive, receptive, or both) and by baseline HSV-2 serostatus (positive or negative). Questions used to assess sexual risk behavior, including those on anal sex preferences, were previously validated by study staff in an HIV sentinel surveillance study conducted with over 2,500 volunteers from Peru and Ecuador [29]. Additionally, a 35-question computer-assisted self-interviewing (CASI) questionnaire was administered to assess tolerability and acceptability of interventional procedures (using a five-point Likert scale), occurrence of social impacts, hygiene patterns, and sexual behavior and satisfaction. The behavioral questions were previously evaluated for terminology and cultural competency through five in-depth interviews and two focus groups of MSM in Lima. Clinic staff was blinded to all CASI answers throughout the study.

At each visit except for week 16, a venous sample for serum (when indicated for STI serology testing), plasma and peripheral blood mononuclear cells (PBMC) was collected.

HIV risk reduction counseling followed local standard procedures, and included the provision of condoms and lubricants [30].Subjects also received counseling on abstinence from certain sexual activities, to help ensure safety after circumcision and rectosigmoid biopsy procedures.

### Interventional Procedures

In this observational study, clinic staff and participants were not blinded to the conduct of the interventional procedures, as all participants were to receive the same procedures and specific safety recommendations were discussed before and after each. Subjects were required to have no RAI nor put anything into their rectum from three days before through seven days after each sigmoidoscopy (with the exception of one Ringer´s lactate enema twelve hours before the procedure). Participants could undergo the procedures if no signs or symptoms of infection were detected on the day of procedure. Flexible sigmoidoscopies were conducted at weeks 2 and 27 by a certified gastroenterologist, after a second Ringer’s lactate enema was performed at an ambulatory surgery clinic. At each procedure, up to 25 sigmoid biopsy samples were collected with a 3.3mm diameter punch (six at 30cm from the anal margin, five at 25cm, six at 20cm, five at 15cm, and three at 10cm). Four additional biopsies were taken from the rectum. Clinical evaluations were performed at weeks 3 and 28 to confirm proper recovery, to assess stool changes, abdominal discomfort, signs and symptoms of bleeding or infection, and to provide medical clearance for resumption of RAI.

Circumcision was conducted by a certified urologist under sterile conditions at the ambulatory surgery clinic at week 4 post enrollment. The skin was checked for signs or symptoms of infection, and if negative, it was prepared with povidone-iodine before administration of local anesthesia via a dorsal penile nerve block with lidocaine and bupivacaine. The foreskin was removed using the sleeve or guided forceps procedures, per urologist discretion. Bleeding was controlled with electrocautery and skin edges were sutured with 4–0 absorbable sutures. Oral antibiotic prophylaxis post-circumcision was prescribed in all cases. Abstinence from masturbation and from insertive sexual activity (receiving oral sex, vaginal intercourse, or insertive anal intercourse [IAI]) was advised for six weeks after circumcision. Clinical evaluations were performed at weeks 5 and 10 to confirm proper healing, document any complications, and specifically at week 10 to provide medical clearance for resumption of insertive sexual activity.

In addition to reimbursement for a participant’s time, study staff facilitated on-time attendance to interventional procedures by providing dedicated transportation to ambulatory surgery facilities. All follow up from the study was concluded by May 2013.

#### Safety laboratory assays

A medical evaluation and hemogram were conducted at each visit. The presence of serum antibodies to HIV-1/2, *Treponema pallidum*, and HSV-2 were assessed at specific study times as described above. HIV diagnosis followed the HVTN algorithm [31]. The earliest date of detectable HIV-1 was determined by plasma HIV-1 RNA in stored samples at University of Washington Virology Laboratory (Seattle, WA). HSV-1/2 infection was diagnosed by a reactive EIA in samples with an index ratio >3.5 to improve the specificity of the assay [32]. Positive and indeterminate serology HSV-1/2 test results were subject to HSV-2 western blot testing at University of Washington Virology Laboratory. Syphilis was diagnosed using RPR-TPHA at IMPACTA. A nucleic acid amplification test was used for the qualitative *in vitro* detection and differentiation of ribosomal RNA of *C*. *trachomatis* and *N*. *gonorrhoeae* at the King County Public Health Laboratories (Seattle, WA). All assays were validated for clinical diagnosis.

#### T-cell phenotypic analysis

PBMC were isolated and cryopreserved following standard procedures [33], then shipped to the FHCRC laboratory in Seattle for later analysis. To evaluate T-cell activation, cryopreserved PBMC were thawed in R10 (RPMI 1640 supplemented with 2mML-glutamine, 25mM HEPES (Invitrogen) 10% FBS (Benchmark), 50μg/ml streptomycin, 50U/ml penicillin (Invitrogen), and 50U/ml benzonase (Novagen), and then washed and stained immediately. Sample viability was determined by LIVE/DEAD Aqua Cell Stain (Invitrogen). Surface staining used integrin β7 Phycoerythrin Cyanine (PECy) 5, Cluster of differentiation (CD) 49d (α4) Allophycocyanin **(**APC), C-C chemokine receptor type 5 (CCR5) PECy7, Cutaneous Leukocyte Antigen (CLA) biotin, CD14 V450 (all Becton Dickinson [BD] Biosciences), and CD103 (αE) Peridinin Chlorophyll Protein Cyanine (PerCPCy) 5.5 (Acris Antibodies). Intracellular staining used CD3 Ax700, Ki-67 Fluorescein Isothiocyanate FITC, B cell lymphoma 2 (Bcl-2) PE, Streptavidin APC-Cy7 (all BD), and CD4 Energy Coupling Dye ECD (Beckman Coulter). Flow cytometric analysis was performed using an LSR II flow cytometer (BD); post-acquisition analysis used FlowJo software (Tree Star, Inc). Personnel involved in thawing, staining and acquiring flow cytometric data were blinded from samples’ pre/post procedure status.

#### Lipopolysaccharide Binding Protein (LPB) quantitation

To facilitate trial conduct, blood was collected on ACD tubes without fasting requirements. Plasma was isolated by two consecutive centrifugations: one at 300×*g* to separate plasma from cells, and a second at 800–1000×*g* to remove heme-containing contaminant. Plasma aliquots were stored at -80°C. Plasma LPB was quantified by ELISA (AntibodiesOnline.com) in the FHCRC laboratory with operators blinded as to pre/post procedure status. According to manufacturer instructions, normal LBP values range from 5–15 μg/ml, with an inter-assay variation coefficiency ranging from 9.8–17.8μg/ml depending on the concentration.

### Data Collection

The privacy of participants was protected by assigning unique identifiers in place of the participant’s name on study data and specimens. In addition, each staff member signed a Confidentiality Agreement with the clinical site agreeing to protect confidential information.

Site staff recorded participant information on clinical research forms (CRFs). Independent data managers conducted quality control for data accurateness and consistency before faxing to HVTN Statistical and Data Managing Center. All study data has verifiable source documentation stored in secured locations.

Additionally, the study site was monitored under contract to the National Institute of Allergy and Infectious Diseases of the U.S. National Institutes of Health.

### Statistical Analyses

Regardless of enrollment group, participant results were analyzed as a single cohort. Descriptive statistics were used to summarize participant characteristics, tolerability and acceptability of interventional procedures, sexual behavior and occurrence of procedure-related events (by MedDRA preferred-term). Fisher’s exact tests were used to compare behavioral differences among dropout and retained participants.

Immune activation data (flow cytometry and LBP) were expressed as medians and interquartile ranges (IQR). Wilcoxon signed rank tests [34] were used to compare pre- and post-procedure immune activation, and participants with and without STIs. To prevent confounding associated with HIV disease process, immune activation data excludes all visits for two participants with confirmed HIV infection. Flow cytometry analysis used 165 PBMC samples, and excluded 3 with viability less than 70% after thawing and staining.

Statistical significance was defined at p<0.05. All analysis used SAS 9.2 (SAS Institute Inc, Cary, NC), and graphs were created using R 2.15.1 (R Foundation for Statistical Computing, Vienna, Austria).

## Results

### Study Enrollment and Participation

Twenty-nine individuals (median age 24 years; all Latino/Hispanic) were enrolled in this cohort (Table 1). Twenty (69.0%) self-identified as homosexual, the rest as bisexual. In the six months preceding screening, participants had a median of eight male sex partners. Twenty-two (75.9%) stated they had practiced RAI with men (eight reported always using condoms) and 23 (79.3%) reported IAI with men (twelve reported always using condoms). Eight (27.6%) reported a female partner (median, 1) in the past six months (three reported using condoms during vaginal sex, two reported using condoms during anal sex). Fifteen participants reported having a steady job; eight reported being students (six with a part-time job); four non-students had part-time employment; and two were unemployed.

**Table 1:** Self-reported Demographic and Sexual Behavior of Study Participants During the Six Months Prior to Screening.*

Characteristics	N (% of total)	Interquartile Range
Median age in years	24	24–26
Median Body Mass Index (BMI)	24	23–26
Self-reported sexual identity		
Homosexual	20 (69.0%)	
Bisexual	9 (31.0%)	
Main sexual role		
Exclusively/mostly insertive	10 (34.4%)	
Half insertive, half receptive	9 (31.0%)	
Exclusively/mostly receptive	10 (34.5%)	
**Sexual behavior with men**	29 (100.0%)	
Median number of sex partners	8.0	3.0–12.0
Insertive oral sex	24 (82.8%)	
Receptive oral sex	23 (79.3%)	
Receptive anal intercourse (RAI)	22 (75.9%)	
RAI always used condoms	8 (36.4% of all RAI)	
Median number RAI partners	5.0	3.0–15.0
Insertive anal intercourse (IAI)	23 (79.3%)	
IAI always used condoms	12 (52.2% of all IAI)	
Median number of IAI partners	5.0	2.0–10.0
**Sexual behavior with women**	8 (27.6%)	
Median number of female partners	1.0	1.0–2.0
Insertive oral sex	6 (75.0%)	
Insertive anal intercourse (IAI)	5 (62.5%)	
IAI always used condoms	2 (40% of all IAI)	
Median number IAI partners	1.0	1.0–2.0
Vaginal sex	8 (27.8%)	
Vaginal always used condoms	3 (37.5% of all vaginal)	
Median number of vaginal sex partners	1.5	1.0–2.0

* Summary enrollment data for 29 study participants

At week 0, HSV-2 infection was documented in 13 participants (44.8%). Syphilis seroreactivity was observed in five participants (17.3%), and in all cases it was diagnosed as a previously treated infection and required no further treatment. Of the 28 participants who underwent STI testing at week 0, three (10.3%) were diagnosed with chlamydia and gonorrhea, four (13.8%) with chlamydia, and one (3.4%) with gonorrhea. Two participants had urethral chlamydia (6.8%) in addition to rectal gonorrhea (n = 1) or rectal gonorrhea and chlamydia (n = 1). Four participants had rectal chlamydia only (13.6%), one had rectal gonorrhea only (3.4%), and one had rectal gonorrhea and chlamydia (3.4%) but no urethral infections. Of these, four participants diagnosed with chlamydia and/or gonorrhea dropped out before receiving diagnosis and treatment. Four received antibiotic treatment during the study period, after their first sigmoidoscopy (n = 4) and circumcision (n = 2) had been conducted. No participant tested positive for chlamydia and/or gonorrhea at week 26 before the second sigmoidoscopy.

At week 26, two participants had reactive HIV-1/2 serology tests. Retrospective testing revealed detectable plasma HIV-1 RNA in one participant at screening, indicating HIV exposure preceding the procedures. The second participant had no HIV-1 RNA detectable in plasma collected at week 10, suggesting he acquired HIV after the period of sexual abstinence post-circumcision. These participants were terminated from HVTN 914 per protocol and referred for HIV management.

### Follow-Up and Tolerability of the Procedures

The main objective of this study was to assess the feasibility of performing safe and tolerable circumcision and rectosigmoid biopsy studies in Lima, Peru. To assess tolerability, we assessed pre- and post-procedure retention throughout the study visits.

Twenty-three participants (79.3%) underwent the first sigmoidoscopy at week 2 (Fig 1).Twenty-one participants (72.4%) completed the circumcision at week 4, including one who completed his circumcision 1 week later than the study plan, in accordance with a planned study deviation. Sixteen participants (55.2%) completed a second sigmoidoscopy at week 27.

**Fig 2:**
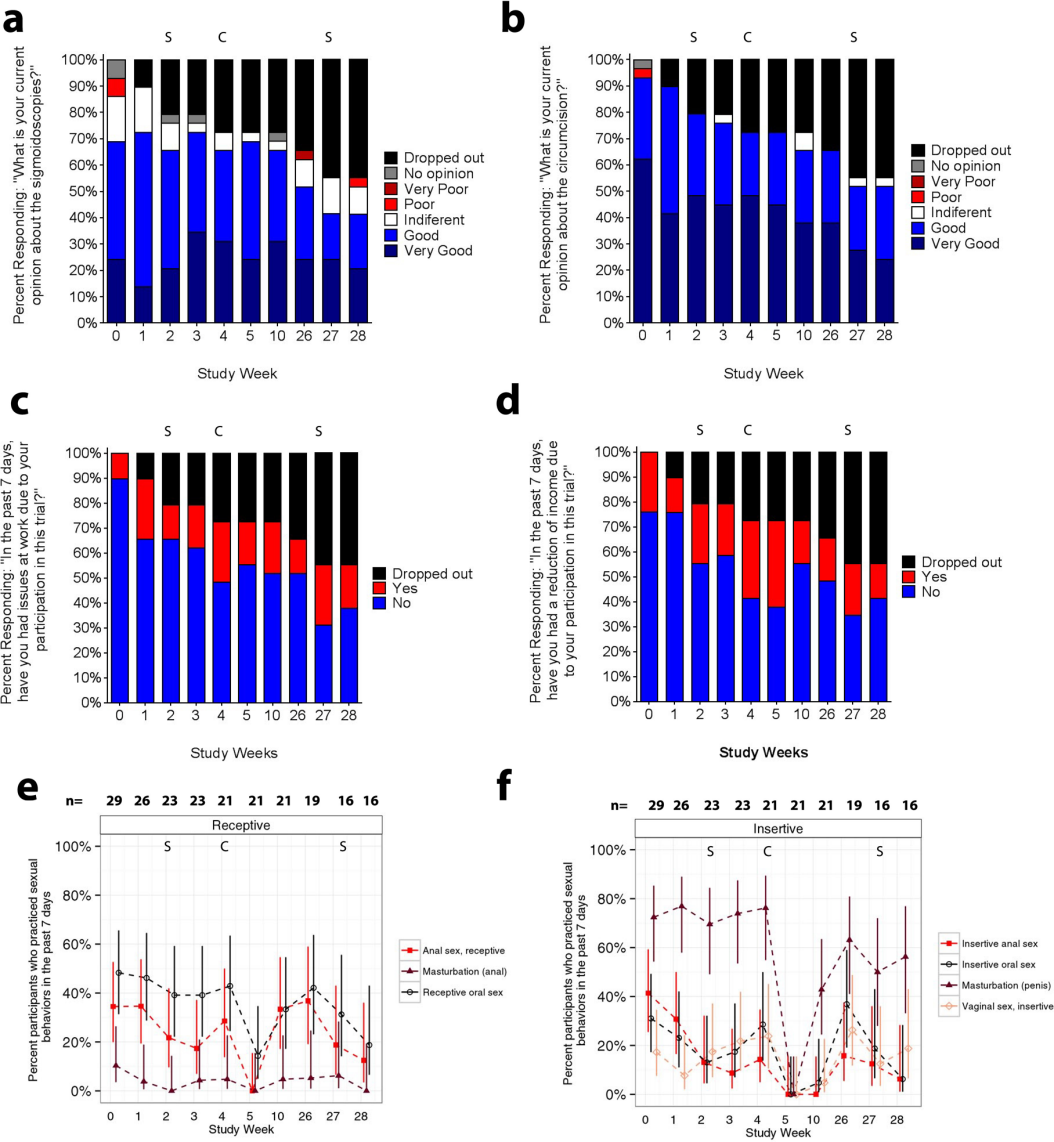
Study tolerability, barriers to study participation, and sexual behaviors according to CASI. A) Opinion of the sigmoidoscopy procedures conducted at weeks 2 and 27. B) Opinion of the circumcision conducted at week 4. C) Perception of loss of work opportunities. D) Perception of reduction of income. E) Receptive anal behaviors (red lines) evaluated abstinence recommendations before and after the sigmoidoscopies at weeks 2 and 27, lasting through weeks 3 and 28 respectively. Receptive oral sex was (black lines) was also monitored but it was not part of abstinence recommendations. F) Insertive penile behaviors evaluated abstinence recommendations after circumcision at week 4, lasting through week 10. In A- F, S marks sigmoidoscopy visit, C indicates the circumcision visit. Confidence intervals were calculated using Wilson scores [48].

Importantly, all participants who underwent any interventional procedures completed the associated post-procedure safety and tolerability evaluations (Fig 1). Retained participants reported very good/good tolerability and acceptance of sigmoidoscopies and circumcision (Fig 2A and 2B). Most participants leaving the study early also reported a good/very good opinion of the procedures at earlier visits (11/13), with only one participant reporting no opinion and another reporting a poor opinion of the sigmoidoscopies.

The most frequent dropout reason cited was difficulty adhering to the procedure schedule because participants had new job opportunities incompatible with convalescence (Fig 1). Some participants associated trial participation with loss of work opportunities (Fig 2C) and reduction in income (Fig 2D). Nevertheless, to examine if abstinence requirements encouraged drop-out, sexual behavior before the procedures (week 0) was compared post-hoc between drop-out and retained participants. There was no evidence that retained participants were less sexually active or more prone to using condoms than those who left the study early (data not shown). However, 91.7% (11/12) of participants who dropped-out had an STI at enrollment or screening visit, whereas only 43.8% (7/16) of those retained were STI-positive (p = 0.0159).

### Procedure Related Events

To assess safety, procedure-related events and hemograms were monitored throughout the study. Clinical evaluations documented adverse drug reactions, edema, hemorrhage/hematoma, infection, pain, perforation or anatomic injury, and erection disabilities. No clinically significant changes in hematology values were observed over time (data not shown). Flexible sigmoidoscopies yielded few post-procedure events; all were mild and resolved on the procedure day (Table 2). Most circumcision-related events presented within the first 9 days post-circumcision (data not shown) and all resolved before study termination. The most common events were pain, edema, and hematoma/hemorrhage at the wound site, which were expected post-operatively and managed accordingly (Table 2).

**Table 2:** MedDRA-coded Procedure Related Events. *

Procedure Related Event Associated with	Mild		Moderate		Severe		Total	
**Sigmoidoscopies**	**Events**	**n (% of 23)**	**Events**	**n (% of 23)**	**Events**	**n (% of 23)**	**Events**	**n (% of 23)**
Adverse Drug Reaction	0	0 (0%)	1	1 (4.3%)	0	0 (0%)	1	1 (4.3%)
Flatulence	1	1 (4.3%)	0	0 (0%)	0	0 (0%)	1	1 (4.3%)
Bloating	1	1 (4.3%)	0	0 (0%)	0	0 (0%)	1	1 (4.3%)
**Circumcision**	**Events**	**n (% of 21)**	**Events**	**n (% of 21)**	**Events**	**n (% of 21)**	**Events**	**n (% of 21)**
Genital Pain	9	7 (33.3%)	5	5 (23.8%)	0	0 (0.0%)	14	12 (57.1%)
Genital Edema	5	5 (23.8)	1	1 (4.8%)	0	0 (0.0%)	6	6 (28.6%)
Genital Hematoma/Hemorrhage	3	3 (14.3%)	2	2 (9.5%)	0	0 (0.0%)	5	5 (23.8%)
Genital Erythema	2	1 (4.8%)	0	0 (0.0%)	0	0 (0.0%)	1	1 (4.8%)
Hypertensive crisis	0	0 (0.0%)	0	0 (0.0%)	1	1 (4.8%)	1	1 (4.8%)
Erectile dysfunction	1	1 (4.8%)	0	0 (0.0%)	0	0 (0.0%)	1	1 (4.8%)
Genital Infection	1	1 (4.8%)	0	0 (0.0%)	0	0 (0.0%)	1	1 (4.8%)
Serous discharge	1	1 (4.8%)	0	0 (0.0%)	0	0 (0.0%)	1	1 (4.8%)

* Columns show the number of MedDRA-coded events and the number of participants affected, who completed a flexible sigmoidoscopies at least once (n = 23) or were circumcised (n = 21). Some participants experienced multiple symptoms at the same time, especially in the first 9 days post-procedure. In a few cases, pain (n = 2) and erythema (n = 1) resolved, re-appeared three months after circumcision, and resolved again, so they were indicated as separate events.

No complications were reported by the six participants who underwent procedures in Lima before receiving their Chlamydia/gonorrhea PCR result (which was only validated in a US laboratory) and treatment. One participant with rectal chlamydia reported pain after the circumcision.

### Adherence to Sexual Abstinence Recommendations

To assess safety of the procedures in a sexually active MSM population, we monitored HIV risk behaviors, and adherence to sexual abstinence recommendations using a CASI questionnaire at each study visit. There was a trend toward a decrease in the proportion of men reporting anal masturbation and RAI with male partners during the seven days pre- and post-sigmoidoscopies when receptive abstinence was advised, compared to the proportion reporting these behaviors outside of this period (Fig 2E). However, 17.4% (4/23 participants) and 12.5% (2/16 participants) continued to have RAI with men after the first and second sigmoidoscopies, respectively.

In contrast, participants did not report any penile masturbation, insertive oral sex, or IAI at week 5 and 10 study visits encompassing the six week post-circumcision abstinence period (Fig 2F). Only one participant of 23 reported insertive oral sex and vaginal intercourse without a condom during the week preceding his week 10 study visit, the last week of the advised post-circumcision abstinence period (Fig 2F).

### Sexual Satisfaction

To further assess tolerability, every participant reported their level of satisfaction with regard to sexual behaviors carried out in the week previous to the clinic visit. In the three weeks previous to the first flexible sigmoidoscopy, 19/24 RAI activities (79.1%) were reported as satisfying or very satisfying. In the four visits posterior to the recommended re-initiation of RAI, 18/20 (90%) RAI events were satisfying or very satisfying. Previous to flexible sigmoidoscopies, 3/4 rectal masturbation activities (75%) were satisfying or very satisfying, whereas 2/3 (66.6%) reported satisfaction after the flexible sigmoidoscopy procedure.

In the five weeks previous to circumcision, 100% of sex with women (21 vaginal events, 9 insertive oral events, and 4 IAI) was reported as satisfying or very satisfying; that percentage did not change after resuming insertive sex post-circumcision (10 vaginal events, 7 insertive oral, and 4 IAI). Of the 25 IAI with men reported before circumcision, 18 were reported as satisfying or very satisfying (72%), whereas 100% (6/6) reported satisfaction with these insertive behaviors in the 3 visits that followed the healing period from circumcision. 17 out of 22 insertive oral sex events with men (77%) reported being satisfied or very satisfied, whereas 6/6 (100%) reported satisfaction in the 3 visits that followed the healing period from circumcision. 81.3–87.5% of individuals reported being satisfied or very satisfied with penile masturbation in the visits previous to the circumcision (n = 16–21), whereas 66.7–75% reported satisfaction in the visits posterior to the abstinence from insertive activities (n = 8–12).

### Systemic Immune Activation

**Fig 3:**
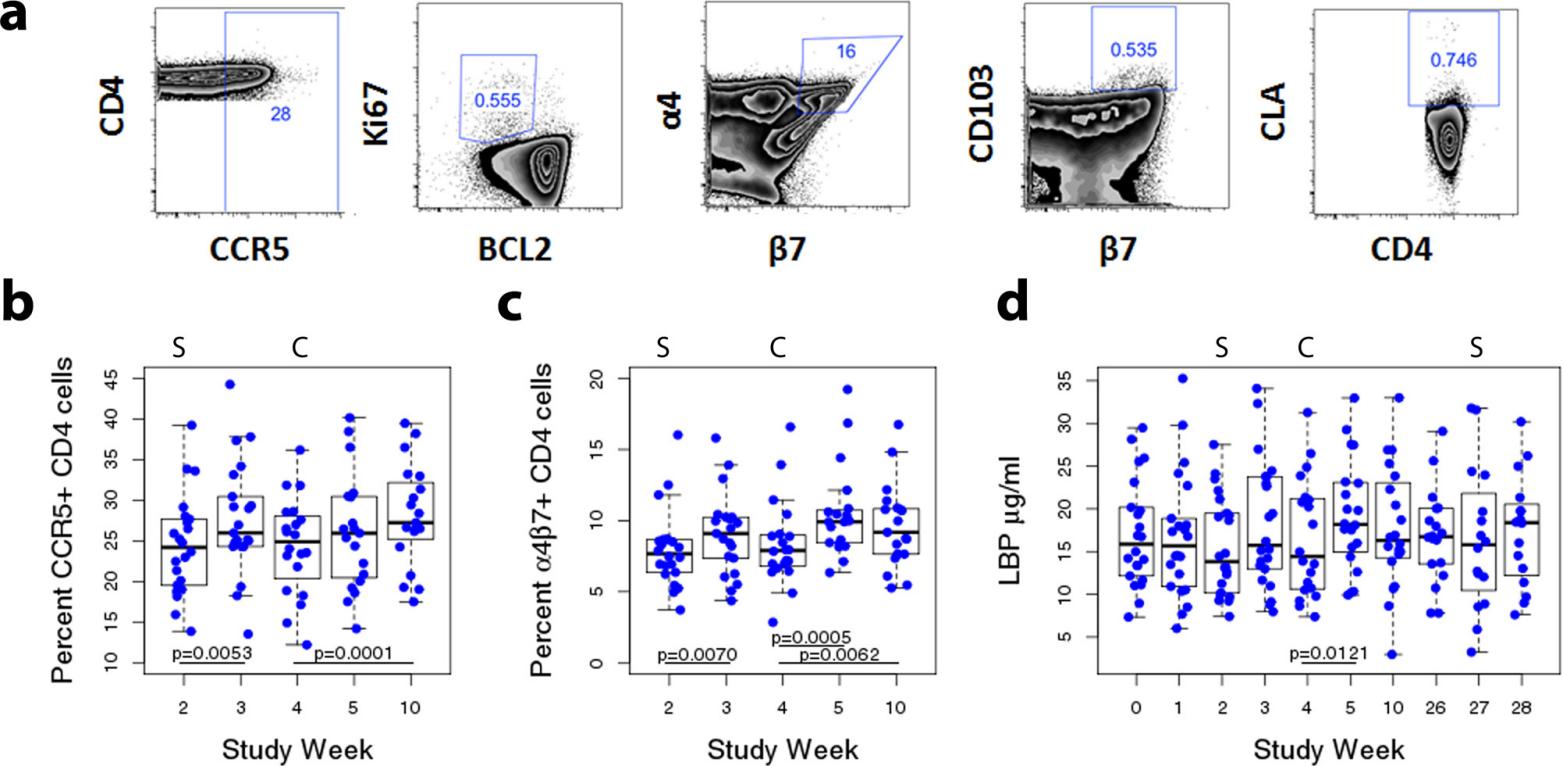
Immune Activation Associated with Interventional Procedures. A) Representative flow cytometry panels indicating live, CD3^+^, CD14^-^, CD4^+^ cells and the gating scheme for CCR5^+^, Ki-67^+^Bcl-2^low^, α4β7^+^, CD103^+^β7^+^, and CLA^+^ cells. 3 samples of 168 were excluded due to cell viability less than 70%.B) Percent CCR5^+^CD4^+^ T cells in blood over time. C) Percent α4β7^+^CD4+ T cells in blood over time. D) LBP concentration in serum over time. In panels B-D, dots indicate individual measurements, with box plots summarizing median and IQR; whiskers represent the range. S marks sigmoidoscopy visit, C indicates the circumcision visit. P-values are from Wilcoxon tests used to examine difference between sigmoidoscopy visits (week 2 and 27) and follow-up at weeks 3 and 28, respectively; only significant values are shown. Similar analysis was conducted comparing the circumcision visit (week 4) with the follow-up period including weeks 5 and 10.

Immune activation of CD4^+^ T cells was evaluated based on expression of CCR5 and Ki-67^+^Bcl2^low^, as these have been associated with high-risk sexual behavior, HIV-1 transmission, and *in vitro* infectivity [35, 36]. Within CD4^+^T cells we also assessed CLA, αEβ7, and α4β7, as surrogates of cell recruitment and migration to genital and intestinal surfaces [37, 38] (Fig 3A and S1 Table). No changes in CLA^+^, αEβ7^+^ or Ki-67^+^Bcl2^low^ CD4^+^ T cells were observed following the sigmoidoscopies (S1 Table). However, the frequency of circulating CCR5^+^CD4^+^ T cells was slightly but significantly elevated one week post-sigmoidoscopy (week 3 median: 26.0%, IQR: 24.3–30.5%) compared to the day of the procedure (week 2 median: 24.2%, IQR: 19.5–27.7%; p = 0.0053; Fig 3B). Similar results were observed before (week 27 median: 23.6%, IQR: 21.2–26.5%) and after the second sigmoidoscopy (week 28 median: 27.3%, IQR: 21.9–29.9%; p = 0.0256; data not shown). A similarly small but significant increase in the frequency of α4β7^+^CD4^+^ T cells was also detected following the first sigmoidoscopy (median week 2: 7.7%, IQR: 6.4–8.7%; median week 3: 9.1%, IQR: 7.4–10.2%; p = 0.0070; Fig 3C). Together the results suggest sigmoidoscopies can lead to small, acute increases in CCR5^+^ and α4β7^+^CD4^+^ T cells in blood, without affecting other markers. No differences in the CCR5^+^CD4^+^ T (p = 1.000) or α4β7^+^CD4^+^ T cells (p = 1.000) were seen among men with STIs and men with negative STI tests.

CCR5^+^CD4^+^T-cell frequencies returned to baseline by two weeks post-sigmoidoscopy (week 4 median: 24.9%, IQR 20.3–28.1%; p = 0.1231; Fig 3B), when others have shown complete healing of the mucosal compartment post-sigmoidoscopies of men practicing RAI [22]. Similar findings were observed among α4β7^+^CD4^+^ T cells (Fig 3C, p = 0.5459). Thus, the transient up-regulation of markers at week 3 returned to baseline within 2 weeks of biopsy.

Circumcision triggered more long-lasting changes in the frequencies of HIV target cells. Compared to week 4, a trend toward increased CCR5^+^CD4^+^ T cells was observed one week post-circumcision (week 5 median: 26.0%, IQR 20.5–30.5%; p = 0.1042), and a significant increase was detected at the end of the recommended abstinence period (week 10 median: 27.2%, IQR: 24.3–33.0%; p = 0.0001; Fig 3B).

Increases in α4β7^+^CD4^+^ T cells were also detected at weeks 5 (median: 9.9%, IQR: 8.4–10.8%; p = 0.0005) and 10 (median: 9.2%, IQR: 7.6–10.9%; p = 0.0062) compared to week 4 (median: 7.9%, IQR: 6.8.-9.0%; Fig 3C). At week 10, there was also a significant increase in Bcl2^low^Ki-67^high^CD4^+^ T cells compared to week 4 (p = 0.0204; S1 Table). No increases in αEβ7^+^or CLA^+^CD4^+^ T cells were observed (S1 Table). All circumcision-associated changes in CD4^+^ T-cell activation remained significant after excluding participants reporting multiple procedure-related events.

Significant increases in LBP, a proposed sensitive acute marker for bacterial infection [39, 40], were detected one week post-circumcision (week 5 median: 18.2μg/ml, IQR: 15.0–23.1μg/ml) when compared to the day of the procedure (week 4 median: 14.4 μg/ml, IQR: 10.6–21.2μg/ml; p = 0.0121; Fig 3D). LBP concentrations did not change after sigmoidoscopies.

## Discussion

This study was designed to be representative of a potential genital and rectal tissue sampling schedule after delivery of an HIV vaccine. Although not powered to assess the incidence of rare procedure-related events, our study suggests that circumcision and sigmoidoscopies can be performed safely in MSM with high-risk sexual behaviors. First, all participants who underwent interventional procedures completed the associated post-procedure evaluations. Second, hematological tests did not show clinically significant signs of blood loss, despite conduct of the procedures simultaneous with phlebotomy. And third, rates of complications such as infection, adverse drug reactions, problems with urination or erectile dysfunction were similar to the 0.5–10% adult male circumcision adverse event rates [20, 41–43] or the 0.004–0.00001% colonoscopy complications [44, 45] reported in the literature. From a safety standpoint, our data demonstrates the feasible implementation of these genital and mucosal collections in MSM at high HIV risk.

Interestingly, our data suggests that circumcision and sigmoidoscopy triggered activation in peripheral HIV target cells, which can be detected throughout the sexual abstinence periods. Systemically, the procedures were followed by an 8.2–15.3% increase in CCR5^+^CD4^+^T cells and a 16.8–32.9% increase in α4β7^+^CD4^+^ T cells compared to pre-procedure samples. As in other elective operations [46], a slight LBP increase was detected post-circumcision. Future studies combining these procedures and peripheral activation endpoints should time systemic assessments outside the procedure recovery window.

Because six participants had asymptomatic HSV-2, rectal chlamydia and/or gonorrhea at the time of their flexible sigmoidoscopies, we considered whether some of the systemic activation could be attributed to the rectal infections. The data argues against this possibility, as there was no difference among the CCR5^+^CD4^+^ and α4β7^+^CD4^+^ T-cell increases among participants with an STI versus those with negative STI tests. Additionally, an increase in CCR5^+^CD4^+^ T cells was observed again after the second sigmoidoscopy, a timepoint where all participants tested negative for the bacterial STIs. We conclude that the changes in peripheral activation can take place in men with or without asymptomatic rectal infections, although in this cohort we were not be powered to study each STI independently.

Peripheral increases in target cells may augment HIV susceptibility [36], Yet, as in studies of medical male circumcision, in this small study, no HIV infections were associated with healing periods with increased T-cell activation [19].

This may reflect how the combination of risk reduction counseling, frequent HIV testing, and abstinence recommendations can support the safe conduct of mucosal sampling in high risk MSM. After circumcision, when the highest increases in immune activation were observed, 20/21 participants reported abstaining from insertive activities for 6 weeks post-procedure, as recommended. Up to 17.4% of participants engaged in receptive anal behaviors within a week of the sigmoidoscopies, suggesting abstinence from receptive behaviors may be more challenging in this MSM population. In fact, receptive sexual behaviors also resumed by week 6 post-circumcision. While overall rates of adherence to insertive abstinence in this trial are consistent with the 78–95% rates seen in recent medical male circumcision trials in Africa [15–17, 19], further work is needed to identify incentives and barriers to compliance with receptive abstinence recommendations in high-risk MSM.

The reasons for differential compliance with insertive versus receptive abstinence are unknown. Visualization of the circumcision wound and penile sensitivity might have discouraged insertive activities during healing, whereas minimal discomfort and no visualization of the intestinal surface may have reduced compliance with post-sigmoidoscopy abstinence. Also, MSM in Lima might exert greater choice over insertive than receptive sexual behaviors [47].

Interestingly, screening data suggests many MSM are willing to undergo invasive procedures as part of an HIV prevention trial. There was a surplus of available volunteers for this trial: 27.8% of eligible volunteers were unable to be enrolled simply based on their HSV-2 sero-positivity or sex role criteria. Our data also reveals a positive evaluation of the circumcision and sigmoidoscopy procedures among the participants who were retained, as well as among the majority (85%) of those who dropped out. We also did not find evidence of changes in sexual satisfaction before the procedures and after resuming sexual activity. However, the low retention prevents us from fully interpreting the tolerability and satisfaction data, as there may be unmeasured barriers to participation in our study design, especially for the men with a previous STI history.

The major reported barriers to participation in the trial were the perceived loss of work opportunities and reduction in income. Repeated post-procedure clinic visits and recovery periods may have been disruptive to employment responsibilities. To provide more flexibility to participants, it will be important to define the kinetics of vaccine and microbicide effects in the mucosa to determine whether wider visit windows could be utilized without compromising mucosal endpoints.

In conclusion, this small study demonstrated that sexually active at-risk MSM in Lima, Peru were willing to participate in a trial of mucosal collections, including circumcision. The study design allowed intensive follow-up and post-procedure counseling and demonstrated the safety of the procedures in this population. Although the clinical implications of the increase in peripheral immune activation markers following the procedures are unknown, this finding reinforces the need for ongoing risk reduction counseling and support for post-procedure abstinence recommendations. This study also generated intriguing hypotheses regarding the acceptability of these procedures and their associated abstinence recommendations, which may inform improved retention efforts in future trials utilizing these procedures in a high-risk MSM population.

## Supporting Information

S1 Checklist TREND Statement checklist. (DOCX)

S1 Protocol HVTN 914: A cohort study in Lima, Peru to evaluate the feasibility of measuring immune responses and activation levels in the foreskin and rectosigmoid mucosa in HIV-negative, uncircumcised men who have sex with men and who are at high risk for HIV acquisition. (PDF)

S1 Table Results from flow cytometry analysis comparing pre- and post-procedure expression of activation and migration markers in peripheral CD4+ T cells. (DOCX)
