# Supplementary material for: Transient Peripheral Immune Activation follows Elective Sigmoidoscopy or Circumcision in a Cohort Study of MSM at Risk of HIV Infection
Source: PLoS One. 2016 Aug 18;11(8):e0160487. doi: 10.1371/journal.pone.0160487 (PMC4990246; doi:10.1371/journal.pone.0160487)
Supplement: S1 Protocol — (PDF) [file pone.0160487.s002.pdf]

HIV VACCINE
TRIALS NETWORK

PROTOCOL

HVTN 914

A cohort study in Lima, Peru to evaluate the feasibility of measuring immune responses and activation levels in the foreskin and rectosigmoid mucosa in HIV-negative, uncircumcised men who have sex with men and who are at high risk for HIV acquisition

DAIDS DOCUMENT ID 11704

STUDY SPONSORED BY

Division of AIDS (DAIDS)
National Institute of Allergy and Infectious Diseases (NIAID)
National Institutes of Health (NIH)
Department of Health and Human Services (DHHS)
Bethesda, Maryland, USA

August 16, 2010
HVTN 914, Version 1.0

This protocol is being provided in support of the manuscript, "Transient peripheral immune activation follows elective sigmoidoscopy or circumcision in a cohort study of MSM at risk of HIV infection" For any other use, please contact template@hvtn.org

Contents

1 Ethical considerations 4

2 IRB/IEC review considerations 6
2.1 Minimized risks to participants 6
2.2 Reasonable risk/benefit balance 6
2.3 Equitable subject selection 6
2.4 Appropriate informed consent 6
2.5 Adequate safety monitoring 7
2.6 Protect privacy/confidentiality 7

3 Overview 8
3.1 Protocol Team 10

4 Background and rationale 11
4.1 Study aims 12
4.2 Study design and study population 12
4.3 Study hypotheses 14

5 Objectives and endpoints 15
5.1 Primary objectives and endpoints 15
5.2 Secondary objectives and endpoints 15
5.3 Exploratory objectives and endpoints 16

6 Statistical considerations 17
6.1 Accrual and sample size calculations 17
6.2 Randomization and blinding 18
6.3 Statistical Analysis 18
6.4 Enrollment stratification monitoring 19

7 Selection and withdrawal of participants 20
7.1 Inclusion criteria 20
7.2 Exclusion criteria 21
7.3 Participant termination from the study 22

8 Clinical procedures 23
8.1 Informed consent 23
8.2 Pre-enrollment procedures 24
8.3 Enrollment and follow-up visits 25
8.4 HIV counseling and testing 26
8.5 STI assessment and treatment 27
8.6 Flexible sigmoidoscopy 27
8.7 Circumcision 27
8.8 Visit windows and missed visits 28
8.9 Early termination visit 28

9 Laboratory 29
9.1 HVTN CRS laboratory procedures 29
9.2 Total blood volume 29
9.3 Mucosal specimen processing and preparation 29
9.4 Immunological assays 29
9.5 Other use of stored specimens 30
9.6 Biohazard containment 31

10 Safety oversight and safety review 32
10.1 HVTN 914 PSRT 32
10.2 SDMC roles and responsibilities in safety monitoring 32
10.3 Safety reporting 33
10.4 Procedure-specific safety criteria assessment 34
10.5 Study termination 34

11 Protocol conduct 35
11.1 Overview of data collection methods 35
11.2 HVTN CRS monitoring 37
11.3 Social impacts 37
11.4 Study participant reimbursement 38

12 Version history 39

13 Document references (other than literature citations) 40

14 Acronyms and abbreviations 42

15 Literature cited 44

Appendix A: Sample informed consent form 47

Appendix B: Table of procedures (for sample informed consent form) 60

Appendix C: Medication information sheet: Sigmoidoscopy 61

Appendix D: Medication information sheet: Lidocaine 62

Appendix E: Medication information sheet: Midazolam 63

Appendix F: Laboratory procedures 64

Appendix G: Procedures at CRS 65

1 Ethical considerations

Multiple candidate HIV vaccines will need to be studied in different populations around the world before a successful HIV preventive vaccine is found. It is critical that universally accepted ethical guidelines are followed at all sites involved in the conduct of these clinical trials and related laboratory studies. The HIV Vaccine Trials Network (HVTN) has addressed ethical concerns in the following ways:

- HVTN studies are designed and conducted to enhance the knowledge base necessary to find a preventive vaccine, using methods that are scientifically rigorous and valid, and in accordance with Good Clinical Practice (GCP) guidelines.

- HVTN scientists and operational staff incorporate the philosophies underlying major codes [1-3], declarations, and other guidance documents relevant to human subjects research into the design and conduct of HIV vaccine clinical trials.

- HVTN scientists and operational staff are committed to substantive community input—into the planning, conduct, and follow-up of its research—to help ensure that locally appropriate cultural and linguistic needs of study populations are met. Community Advisory Boards are required by DAIDS and supported at all HVTN research sites to ensure community input.

- HVTN study staff members counsel each participant at each study visit on how to reduce HIV risk. Participants who become HIV-infected during the study are provided counseling on notifying their partners and about HIV infection according to local guidelines. Staff members will also counsel them about reducing their risk of transmitting HIV to others.

- The HVTN requires that all international HVTN sites lacking national plans for providing antiretroviral therapy (ART) develop plans for the care and treatment of participants who acquire HIV infection during a trial. Each plan is developed in consultation with representatives of host countries, communities from which potential trial participants will be drawn, sponsors, and the HVTN. Participants will be referred to programs for ART provision when the appropriate criteria for starting ART are met. If a program is not available at a site and ART is needed, a privately established fund will be used to pay for access to treatment to the fullest extent possible.

- The HVTN provides training so that all participating sites similarly ensure fair participant selection, protect the privacy of research participants, and obtain meaningful informed consent. During the study, participants will have their wellbeing monitored and, to the fullest extent possible, their privacy protected. Participants may withdraw from the study at any time.

- Prior to implementation, HVTN trials are reviewed rigorously by scientists who are not involved in the conduct of the trials under consideration.

- HVTN studies are reviewed by local and national regulatory bodies and are conducted in compliance with all applicable national and local regulations.

- The HVTN designs its research to minimize risk and maximize benefit to both study participants and their local communities. For example, HVTN protocols provide enhancement of participants' knowledge of HIV and HIV prevention, as well as counseling, guidance, and assistance with any social impacts that may result from research participation. HVTN protocols also include careful medical review of each research participant's health conditions and reactions to study products and/or procedures while in the study.

- HVTN research aims to benefit local communities by directly addressing the health and HIV prevention needs of those communities and by strengthening the capacity of the communities through training, support, shared knowledge, and equipment. Researchers involved in HVTN trials are able to conduct other critical research in their local research settings.

- The HVTN recognizes the importance of institutional review and values the role of in-country Institutional Review Boards (IRBs) and Independent Ethics Committees (IECs) as custodians responsible for ensuring the ethical conduct of research in each local setting.

2 IRB/IEC review considerations

US Food and Drug Administration (FDA) and other US federal regulations require IRBs or IECs to ensure that certain requirements are satisfied on initial and continuing review of research (Title 45, Code of Federal Regulations (CFR), Part 46.111(a) 1-7; 21 CFR 56.111(a) 1-7). The following section highlights how this protocol addresses each of these research requirements. Each HVTN Investigator welcomes IRB/IEC questions or concerns regarding these research requirements.

2.1 Minimized risks to participants

45 CFR 46.111 (a) 1 and 21 CFR 56.111 (a) 1: Risks to subjects are minimized.

This protocol minimizes risks to participants by (a) correctly and promptly informing participants about risks so that they can join in partnership with the researcher in recognizing and reporting harms; (b) respecting local/national blood draw limits; (c) having staff properly trained in administering study procedures that may cause physical harm or psychological distress, such as blood draws, HIV testing and counseling, and HIV risk reduction counseling; (d) providing HIV risk reduction counseling; and (e) providing safety monitoring.

2.2 Reasonable risk/benefit balance

45 CFR 46.111 (a) 2 and 21 CFR 56 (a) 2: Risks to subjects are reasonable in relation to anticipated benefits, if any, to subjects, and the importance of the knowledge that may reasonably be expected to result.

In all public health research, the risk-benefit ratio may be difficult to assess because the benefits to a healthy participant are not as apparent as they would be in treatment protocols, where a study participant may be ill and may have exhausted all conventional treatment options. However, this protocol is designed to minimize the risks to participants while maximizing the potential value of the knowledge it is designed to generate.

2.3 Equitable subject selection

45 CFR 46.111 (a) 3 and 21 CFR 56.111 (a) 3: Subject selection is equitable

This protocol has specific inclusion and exclusion criteria for investigators to follow in admitting participants into the protocol. Participants are selected because of these criteria and not because of positions of vulnerability or privilege. Investigators are required to maintain screening and enrollment logs to document volunteers who screened into and out of the protocol and for what reasons.

2.4 Appropriate informed consent

45 CFR 46.111 (a) 4 & 5 and 21 CFR 56.111 (a) 4 & 5: Informed consent is sought from each prospective subject or the subject’s legally authorized

representative as required by 45 CFR 46.416; informed consent is appropriately documented as required by 45 CFR 46.417

The protocol specifies that informed consent must be obtained before any study procedures are initiated and assessed throughout the trial (see section 8.1). Each site is provided training in informed consent by the HVTN as part of its entering the HVTN. The HVTN requires a signed consent document for documentation, in addition to chart notes or a consent checklist.

2.5 Adequate safety monitoring

45 CFR 46.111 (a) 6 and 21 CFR 56.111 (a) 6: There is adequate provision for monitoring the data collected to ensure the safety of subjects.

This protocol has extensive safety monitoring in place (see section 10). Safety is monitored daily by clinical affairs staff and routinely by the Protocol Safety Review Team (PSRT).

2.6 Protect privacy/confidentiality

45 CFR 46.111 (a) 7 and 21 CFR 56.111 (a) 7: There are adequate provisions to protect the privacy of subjects and maintain the confidentiality of data.

Privacy refers to an individual's right to be free from unauthorized or unreasonable intrusion into his/her private life and the right to control access to individually identifiable information about him/her. The term "privacy" concerns research participants or potential research participants as individuals whereas the term "confidentiality" is used to refer to the treatment of information about those individuals. This protocol respects the privacy of participants by informing them about who will have access to their personal information and study data (see Appendix A, section 13). The privacy of participants is protected by assigning unique identifiers in place of the participant's name on study data and specimens. In addition, each staff member at each study site in this protocol signs a Confidentiality Agreement with the HVTN and each study site participating in the protocol is required to have a standard operating procedure on how the staff members will protect the confidentiality of study participants.

3 Overview

Title

A cohort study in Lima, Peru to evaluate the feasibility of measuring immune responses and activation levels in the foreskin and rectosigmoid mucosa in HIV-negative, uncircumcised men who have sex with men and who are at high risk for HIV acquisition

Primary objective(s)

- To assess the feasibility of performing safe and tolerable circumcision and rectosigmoid biopsy studies in Lima, Peru
- To assess institutional capacities to process mucosal samples
- To identify methods of evaluating foreskin and rectosigmoid mucosal immune responses that provide minimal variability for analysis of small sample sizes

Participants

30 healthy, HIV-seronegative, uncircumcised men, aged 21 to 30 years, who have sex with men (MSM) and who are at high risk for acquisition of HIV

Design

Single site, exploratory cohort study

Duration per participant

Approximately 7 months per participant

Estimated total study duration

96 weeks (includes enrollment and follow-up)

Core operations

HVTN Vaccine Leadership Group/Core Operations Center, Fred Hutchinson Cancer Research Center (FHCRC) (Seattle, Washington, USA)

Statistical and data management center (SDMC)

Statistical Center for HIV/AIDS Research and Prevention (SCHARP), FHCRC (Seattle, Washington, USA)

Endpoint assay laboratories

FHCRC (Seattle, Washington, USA)

Asociación Civil Impacta Salud y Educación (IMPACTA) (Lima, Peru)

King County Public Health Department (Seattle, Washington, USA)

Study sites

Miraflores Clinic, Asociación Civil Impacta Salud y Educación (Lima, Perú)

Safety monitoring

HVTN 914 PSRT

4 Background and rationale

Mucosal responses are the first line of defense against HIV infection that is acquired through sexual contact. Multiple randomized clinical trials have indicated that the foreskin plays a role in increased risk of HIV acquisition in heterosexual men [4-6], but the biological mechanisms for HIV risk at the foreskin have not yet been well characterized [7-10]. Among MSM, receptive sexual behavior is also associated with risk of infection [11-14], but the characteristics of the healthy rectosigmoid mucosa have not been fully evaluated or correlated with HIV risk. This study evaluates several biological characteristics of the mucosa of MSM at risk of HIV infection, including their anatomical properties, the density of HIV target cells, and the HIV target cells' proximity to the mucosal epithelium.

Most studies of foreskin mucosa published to date have focused on samples collected from neonates, or from adults with balanitis and phimosis. Multiple caveats must be applied in interpreting the data of these studies. Neonatal foreskin differs from that of sexually active men in keratin thickness and in immune infiltration [15]. Chronic infection in adults requiring circumcision increases the T-cell population at the site, depletes Langerhans cells (LC) from the epithelium, and causes further keratinization of the mucosal surfaces [15,16]. This study is unique in that it will describe the mucosa of healthy, uncircumcised, sexually active individuals at risk of HIV infection.

A few studies have addressed the potential mechanisms of HIV infection in the human vaginal and intestinal mucosa, but none has studied both adult foreskins and rectosigmoid biopsies. This study establishes a baseline for mucosal studies in MSM at risk, addresses variability in these mucosal samples, and assesses the feasibility of incorporating these intensive studies into future HIV vaccine trials.

Vaccine development has aimed to improve mucosal responses, but currently it lacks the tools to study whether vaccination induces correlates of protection at sites of infection. This study aims to evaluate the applicability of several immune assays in mucosal samples in order to determine if these assays could aid vaccine research and could be used to measure mucosal immune responses at sites of HIV exposure.

The relevance of studying mucosal responses in vaccine design was highlighted in the Step Study, which aimed to assess HIV infection risk among MSM participants receiving either placebo or an adenovirus type 5 (Ad5) vector vaccine (MRKAd5 *gag/pol/nef*). Disappointingly, the vector vaccine failed to prevent HIV infection or to reduce the viral set-point at three months after HIV infection [17]. Although the vaccine enhanced T-cell responses against HIV and Ad5 in blood, the interim analysis indicated that participants who received the vaccine were at increased risk of HIV infection compared to participants who received the placebo. Further analysis indicated that vaccinated uncircumcised Ad5-seropositive individuals were at greatest risk [18].

The biological interactions between vaccination and circumcision are not well understood. It has been hypothesized that the increased incidence of HIV infection among Ad5 seropositive vaccine recipients was due, in part, to increased HIV target cells generated at mucosal sites. This study evaluates methods for measuring mucosal immune responses and assesses whether small studies can characterize vaccine-specific T-cell responses at mucosal sites to inform future vaccine trials.

4.1 Study aims

The overarching goal of this study is to explore several measures of mucosal immunity in foreskin and rectosigmoid biopsies. The study focuses upon characterizing the immune response at the mucosal surfaces of the foreskin, sigmoid colon, and rectum because of the strong association between receptive anal sex and HIV infection, and between uncircumcised status and HIV infection [4-6,11,17,18]. In particular, this study aims:

1. To address the feasibility of carrying out mucosal studies in Lima, Peru. The study aims to describe the standards applied to mucosal studies conducted in Lima and to demonstrate that the HVTN has sufficient organizational, operational, and logistical capacity to study mucosal samples at an international site.

2. To establish methods for assessing baseline characteristics of the mucosa of MSM at risk of HIV infection. The study aims to describe the anatomical features of the mucosal surfaces (keratinization and homeostasis), characterize the density, location and migration of HIV target cells at these sites, and quantitate the expression of factors involved in reducing HIV infection risk at the foreskin and lower intestinal mucosa.

3. To evaluate several mucosal assays and determine which assays can provide informative endpoints for future vaccine studies. This study aims to characterize the variability of the endpoints in aim 2 (above) in the mucosa of the MSM population in Lima, determine their change over time, and explore the impact of sexually transmitted infection (STI) history and sexual practices on the endpoints discussed in aim 2 (above).

4. To correlate blood responses, mucosal responses at the foreskin, and mucosal responses at the rectum and sigmoid colon of MSM at risk of HIV. This will determine if an assessment of mucosal responses is essential, or whether assays using peripheral blood mononuclear cells (PBMC) provide sufficient correlates of the mucosal response at sites of HIV exposure.

4.2 Study design and study population

This is a cohort study of foreskin and lower intestinal mucosa from HIV-negative, uncircumcised MSM in Lima, Peru who are at high risk of HIV infection and who practice primarily insertive, versatile (ie, both receptive and insertive), or primarily receptive anal sex (see Table 4-1). Peru provides an excellent setting for circumcision trials, given the high willingness to participate. Among 2048 high risk MSM respondents in a sentinel surveillance survey conducted in 2006 in four Andean Region cities, circumcision prevalence was 3.7%, while 54.3% of uncircumcised men stated they were willing to participate in a circumcision trial. The highest prevalence of circumcision (5.5%) was observed in Lima, where among the 622 uncircumcised participating men, 63.7% showed willingness to be part of a circumcision trial. Willingness was higher, but not significantly so, among those who tested HIV positive and had a sexual insertive role. Principal fears about participation in such a trial were undergoing surgery (45.5%), side effects related to surgery (47.2%), and fear that the partner would insist upon having sex without condoms (43.5%) [19].

As T-cell recruitment to the skin and the level of keratinization of the foreskin varies with age [15], only volunteers in the relatively narrow age range of 21 to 30 years old will be enrolled. This age range was selected because it corresponds to the population of MSM in Lima that appears to have the highest HIV incidence [20], and that represents the majority of participants volunteering in HIV vaccine trials in Lima and other international HVTN clinical research sites (CRSs).

As HVTN 914 aims to establish the feasibility of collecting baseline and postvaccination biopsies in the future, retention in this study with two mucosal biopsy timepoints is monitored.

Among the MSM population in Lima, herpes simplex virus type 2 (HSV-2) prevalence was measured at 49.0% in 2002 [21], whereas syphilis prevalence was measured at 14.3%. Among high risk MSM in Lima, the prevalence of syphilis was 12.4% (rapid plasma reagin [RPR] reactive > 1/1 and microhemagglutination for *Treponema pallidum* [MHA-TP] confirmed) and 3.4% had evidence of active disease (RPR ≥ 1/16 and MHA-TP confirmed) in 2002 [21]. A recent study found that among HIV-seroconverter MSM, 10% tested positive for *Chlamydia trachomatis (*CT) in saliva, urethra, or rectum using a polymerase chain reaction (PCR)-based assay [22]. Thus, it is important to address the potential confounding effects of these infections in our assessment of mucosal inflammation in the study population.

As depicted in Table 4-1, the HVTN 914 study population is stratified on HSV-2 serostatus, to account for the HSV-2–specific immune cell infiltration that is reported to last at least 12 weeks post-healing of visible sores [23,24]. Information about infection with syphilis and other STIs will also be collected and potential correlations between the presence of these infections, keratinization, and accumulation of HIV target cells will be assessed.

Table 4-1 Study population by sexual role preference and HSV-2 status

Sexual role preference for anal sex	Baseline HSV-2 status	
	+	–
Insertive	5	5
Versatile	5	5
Receptive	5	5

The study population is uncircumcised, HIV-seronegative, consenting, and counseled MSM who practice either primarily receptive, versatile, or primarily insertive anal sexual behavior. Because HVTN 914 aims to analyze genital mucosal correlates of HIV infection risk, only sexually active participants are eligible for enrollment. Men involved in transactional sex (sex in exchange for money, drugs, shelter, or food) are excluded, as socioeconomic difficulties with adherence to abstinence recommendations in this population may increase their vulnerability to resuming sex before complete healing from sigmoidoscopies and/or circumcision.

In the 2006 HIV sentinel surveillance conducted in Lima, among high-risk men who reported not knowing their HIV serostatus or not having HIV testing during the previous 12 months but with evidence of high risk behavior for acquiring HIV-1 infection, 492 (32.0%) of 1586 reported being exclusively insertive in their sexual role during the last 5 years, 197 (12.8%) mainly insertive, 328 (21.3%) insertive half of the time and receptive

half of the time, 262 (17.0%) mainly receptive, and 261 (17.0%) exclusively receptive [20]. Thus, the Lima, Peru CRS has the capacity to identify and recruit men reporting different sexual roles.

To mirror the Lima MSM population participating in vaccine trials, in this study we aim to recruit volunteers with different sexual preferences, and examine how these practices affect the variability of study endpoints. In addition, we will evaluate the impact of self-reported sexual behavior on protocol compliance and on retention, in order to understand potential biases in future vaccine studies that collect mucosal samples in a diverse MSM population.

Computer-assisted self-interviews (CASI) will be used at screening and follow-up visits in order to decrease social desirability bias and reporting bias for potentially sensitive and embarrassing questions about participants' specific sexual behavior, function, and satisfaction. There are no data in the literature regarding the potential impact of either circumcision or sigmoidoscopies on sexual behavior, function, and satisfaction among adult MSM. All data known come from the heterosexual male population [25]. For this reason, results from this study will help guide implementation of these procedures in future HIV vaccine trials. Computer-illiterate participants will be assisted by study personnel in completing the CASI.

4.3 Study hypotheses

4.3.1 Primary hypothesis

It is feasible and informative to conduct small cohort studies that assess immunological responses in rectosigmoid and foreskin mucosa collected in Lima, Peru.

4.3.2 Secondary hypotheses

Mucosal immune responses at the foreskin and colon differ from each other and do not correlate with responses observed in PBMCs.

A history of HSV-2 infection and/or a history of bacterial STIs, alters the characteristics of the rectosigmoid and foreskin mucosa, to favor HIV infection of target cells.

4.3.3 Tertiary hypothesis

Role preferences for anal sex (receptive, versatile, insertive) modify the immune responses at the rectosigmoid and foreskin mucosa.

5 Objectives and endpoints

5.1 Primary objectives and endpoints

Primary objective 1:

To assess the feasibility of performing safe and tolerable circumcision and rectosigmoid biopsy studies in Lima, Peru

Primary endpoints 1*:*

Pre- and post-procedure retention, safety laboratory values, sexual satisfaction, HIV risk behaviors, and levels of activation markers associated with vulnerability to HIV infection in PBMC samples; and procedure-related events

Primary objective 2:

To assess institutional capacities to process mucosal samples

Primary endpoints 2*:*

Operational, clinical, and laboratory protocol deviations per mucosal sample collected in Lima and proportion of samples that are evaluable

Primary objective 3:

To identify methods of evaluating foreskin and rectosigmoid mucosal immune responses that provide minimal variability for analysis of small sample sizes

Primary endpoints 3*:*

Inter-person variability in the Lima MSM population in: the anatomical characteristics of the rectosigmoid and foreskin mucosa (keratinization, homeostasis); the density, activation and location of HIV target cells of the foreskin and rectosigmoid mucosa by immunofluorescence microscopy and flow cytometry; expression of innate resistance factors within the foreskin and rectosigmoid mucosa by real-time PCR and antibody-based detection; and antigen-specific T-cells within the foreskin and rectosigmoid mucosa by intracellular cytokine staining (ICS). Intra-person differences in mucosal responses obtained from the rectosigmoid colon at the two timepoints.

5.2 Secondary objectives and endpoints

Secondary objective 1:

To characterize mucosal immune responses in PBMCs, foreskin, and rectosigmoid mucosa

Secondary endpoints 1*:*

Density, activation, and location of HIV target cells in foreskin and rectosigmoid mucosa by immunofluorescence microscopy and flow cytometry; expression of innate resistance

factors within the foreskin and rectosigmoid mucosa by real-time PCR and antibody-based detection; antigen-specific T-cells within the foreskin and rectosigmoid mucosa evaluated by ICS

Secondary objective 2:

To assess the impact of HSV-2 status and/or bacterial STIs on small cross-sectional studies of mucosal immune responses

Secondary endpoints 2*:*

Associations of HSV-2 serology, CT/NG PCR in urine and in rectal secretions, and syphilis testing with anatomical characteristics of the rectosigmoid and foreskin mucosa (keratinization, homeostasis); density, activation, migration and location of HIV target cells in the foreskin and rectosigmoid mucosa by immunofluorescence microscopy and flow cytometry; expression of innate resistance factors within foreskin and rectosigmoid mucosa by real-time PCR and antibody-based detection; and antigen-specific T-cells within the foreskin and rectosigmoid mucosa by ICS

5.3 Exploratory objectives and endpoints

Exploratory objective 1:

To assess the HIV risk associated with sigmoidoscopy and circumcision procedures in sexually active MSM.

Exploratory endpoints 1:

Pre- and post-procedure (1) HIV risk behaviors and (2) levels of activation markers in PBMCs that are associated with vulnerability to HIV infection.

Exploratory objective 2:

To assess the impact of MSM sexual role preferences on immune responses within small cross-sectional studies

Exploratory endpoints 2:

Correlation between sexual preferences (self-identification as insertive, receptive, or versatile) with the anatomical characteristics of the rectosigmoid and foreskin mucosa (keratinization, homeostasis); the density, activation, migration and location of HIV target cells of the foreskin and rectosigmoid mucosa by immunofluorescence microscopy and flow cytometry; expression of innate resistance factors within the foreskin and rectosigmoid mucosa by real-time PCR and antibody-based detection; and antigen-specific T-cells within the foreskin and sigmoid mucosa by ICS

6 Statistical considerations

6.1 Accrual and sample size calculations

This cohort study will enroll 30 HIV-seronegative, uncircumcised 21-30 year old MSM at high risk of HIV infection in Lima, Peru. These subjects will be equally stratified into 6 groups defined by primary sexual preferences (insertive, versatile, or receptive) and baseline HSV-2 status (positive or negative). Participants withdrawing from the study prior to completion of all study procedures planned for week 4 will be replaced with new eligible volunteers belonging to the same stratification group. However, all data from enrolled participants, including those terminated from the study early, will be included in the evaluation of all study objectives, when appropriate.

6.1.1 Sample size calculations for feasibility

One of the goals of the feasibility evaluation for this study is to identify procedure-related events associated with circumcision. Sample size calculations for this study endpoint are expressed in terms of the true event rate above which at least 1 event would likely be observed and the true event rate below which no events would likely be observed. Specifically, for a group of 15 subjects in each of the two strata defined by the baseline HSV-2 status, there is a 90% chance of observing at least 1 event if the true rate of such an event is 15% or more; and there is a 90% chance of observing no events if the true rate is 0.69% or less. For a study with a sample size of 30, there is a 90% chance of observing at least 1 event if the true rate of such an event is 8% or more; and there is a 90% chance of observing no events if the true rate is 0.35% or less.

Table 6-1 Probability of observing 0 events, 1 or more events, 2 or more events, 3 or more events, and 4 or more events among all participants (n = 30) for different true event rates

True event rate (%)	Pr(0/30)	Pr(1+/30)	Pr(2+/30)	Pr(3+/30	Pr(4+/30)
1	0.740	0.260	0.036	0.003	< 0.001
3.5	0.343	0.657	0.283	0.086	0.020
5	0.215	0.785	0.446	0.188	0.061
10	0.042	0.958	0.816	0.589	3.353
20	0.001	0.999	0.989	0.956	0.877
30	< 0.001	1.000	1.000	0.998	0.991

6.1.2 Sample size calculations for measurement of immune responses

The proposed studies are exploratory in nature. Pilot experiments were conducted to determine the variability in many of the immune assays utilized in HVTN 914. Given the importance of assessing the impact of HSV-2 infections on immune responses and activation levels in the study population, sample size calculations for measurement of immune responses are based on observed data from previous studies that have reported differences between the density of immune cells (LCs) at the foreskin of subjects with and without history of infection [15,16], where reported values suggest differences of 30% - 50% [15], and between the density of immune cells at sites with and without HSV-2 infection from the same individual, where reported values suggest differences of 50% - 75% [24]. Table 6-2 presents the statistical power to detect various sizes of differences in

the density of immune cells using groups of 10 and 15 subjects. For example, with 10 men per group, there is around 80% power to detect a reduction of 70% LCs between two groups. If the expected percentage of reduction is less than 55%, then at least 15 subjects per group will be required to achieve 80% power. These calculations assumed that the density of immune cells follows a normal distribution with a mean of 100 cells/mm^2 for HSV-2–infected individuals and a standard deviation of 50 in HSV-2–infected and uninfected subjects. Note that, as reported by Qin et al in 2009, samples with infectious history had 132.2 LCs/mm^2 ± 30 , whereas samples lacking infection history had 87.5 LCs /mm^2 ± 17.6 [15]. Also, as reported by Zhu et al in 2009, the observed mean ± standard deviation (sd) were 80 CD4+ cells/mm^2 ± 41, 127 CD8+ cells/mm^2 ± 71, and 104 CD209+ cells/mm^2 ± 52 [24]. One-sided Wilcoxon rank sum tests with a Type I error rate of 0.05 were used.

Table 6-2 Statistical power (%) to detect various % of reduction in the frequency of immune cells between subjects with and without HSV-2 infection based on sample sizes of n = 10 or 15

% of reduction in the density of immune cells	n = 10	n = 15
40%	36	53
50%	51	71
**55%**	59	**79**
60%	68	86
65%	74	91
**70%**	**79**	**94**

6.2 Randomization and blinding

No randomization and blinding will be implemented in this study.

6.3 Statistical Analysis

Due to the exploratory nature of this study, no formal multiple comparison adjustment will be employed for safety endpoints or immune response endpoints. All descriptive and inferential statistical analyses will be performed using SAS, StatXact, Splus, and/or R statistical software.

6.3.1 Analysis variables

The analysis variables consist of pre- and post-procedure participant characteristics, feasibility indices (including retention, safety laboratory values, procedure-related events, sexual satisfaction, HIV risk behavior, and activation markers), and immune responses. Analysis variables will also include operational, clinical, and laboratory protocol deviations.

6.3.2 Analysis methods

Descriptive analyses will be used to summarize participant characteristics, various feasibility indices, protocol deviations, and various immune responses measured by different methods. The probability of observing a certain number of grade 3 or higher procedure-related events will be assessed to facilitate evaluation of feasibility, given true event rates ranging from 1–5% [4-6]. Rank-based nonparametric tests for paired data (eg, Wilcoxon signed rank tests) will be used to examine intra-person differences of various immune responses between the 2 sigmoidoscopy timepoints. In addition, for a continuous immune response variable, the difference between HSV-2 positive and negative strata will be tested with a non-parametric Wilcoxon rank sum test if the data are not normally distributed and with a two-sample t-test if the data appear to be normally distributed. Analysis of Variance (ANOVA) will also be employed to investigate the possible joint effect of sexual role preferences and HSV-2 serostatus on various immune outcomes. For a categorical immune response variable, generalized linear regression models will be used. Transformation of analysis variables may be needed to satisfy distributional assumptions in these analyses. All statistical tests will be 2-sided and will be considered statistically significant if $p \leq 0.05$.

6.4 Enrollment stratification monitoring

At week 70 following enrollment of the first study participant, the HVTN 914 protocol team will review accrual into each stratification group. If the number of participants in any stratification group is fewer than 3 participants at that time, the protocol team may choose to modify the study design. For instance, the study design may be revised to ensure equal numbers of HSV-2 seropositive and seronegative participants (n = 15) regardless of the sexual role preference of eligible volunteers. Any proposed study modification will be subject to standard review processes for study modifications, including but not limited to DAIDS regulatory review and local IRB/IEC review.

7 Selection and withdrawal of participants

Participants will be sexually active, HIV-seronegative, uncircumcised MSM who comprehend the purpose, procedures, and safety policies of the study and have provided written informed consent. Participants will be stratified into three categories according to sexual role preference during anal sex during the 6 months prior to screening (exclusively insertive, versatile [ie, both insertive and receptive], and exclusively receptive) based on self-report at the screening visit.

Investigators should always use good clinical judgment in considering a volunteer's overall fitness for trial participation. Some volunteers may not be appropriate for enrollment even if they meet all inclusion/exclusion criteria. Medical, psychiatric, occupational, or other conditions may make evaluation of safety difficult, and some volunteers may be poor candidates for retention.

Determination of eligibility, taking into account all inclusion and exclusion criteria, must be made within 56 days prior to enrollment unless otherwise noted in sections 7.1 and 7.2. Note that HSV-2 testing will be conducted at screening for purposes of sample stratification, though it is not an eligibility criterion and is, therefore, not included in sections 7.1 and 7.2. As indicated in section 8.2, this test is not subject to the 56-day screening window. Volunteers will be recruited and screened; those determined to be eligible, based on the inclusion and exclusion criteria, will be enrolled in the study and followed for a period of 28 weeks. Final eligibility determination will depend on results of laboratory tests, medical history, physical examinations, and answers to self-administered and/or interview questions.

7.1 Inclusion criteria

1. **Male,** age 21 to 30 years, who, in the 6 months prior to screening, experienced 1 or both of the following HIV risk criteria:

    - unprotected anal intercourse with 1 or more male or MTF transgender partner(s); or

    - anal intercourse with 2 or more male or MTF transgender partners.

    Note: Volunteers who have been in a monogamous relationship with an HIV-seronegative partner for > 6 months are excluded.

2. Ability and willingness to provide **informed consent**

3. **Assessment of understanding:** volunteer demonstrates understanding of the procedures and purpose of this study. Participants will complete a questionnaire prior to enrollment with verbal demonstration of understanding of all questionnaire items answered incorrectly.

4. Willingness to receive **HIV test results**

5. Willingness to discuss **HIV infection risks** (including sexual behavior and drug use) and amenable to **HIV risk reduction counseling**

6. Willingness to undergo **phlebotomy, rectal swab, sigmoidoscopy, and circumcision**

7. **Willingness to adhere to safety protocols** before and after sigmoidoscopy and circumcision

8. **Agrees not to enroll in another study** of an investigational research agent prior to completion of the last required protocol clinic visit

9. **Hemoglobin** ≥ 13.0 g/dL

10. **White blood cell (WBC) count** = 3300 to 12,000 cells/mm^3

11. **Total lymphocyte count** ≥ 800 cells/mm^3

12. **Remaining differential** either within institutional normal range or with site physician approval

13. **Platelets** = 125,000 to 550,000/mm^3

14. **Prothrombin time (PT)** or **partial thromboplastin time (PTT)** ≤ 1.25 institutional upper limit of normal; **International Normalized Ratio (INR)** ≤ 1.5

15. **Negative HIV-1 and -2 blood test:** May use locally available assays that have been approved by HVTN Laboratory Operations.

7.2 Exclusion criteria

1. **Volunteers who, in the six months prior to screening, have had sexual partners known to be HIV-infected**

2. **Volunteers who, for the 6 months prior to screening,** have been in a monogamous relationship with an HIV-seronegative partner

3. **History of immunodeficiency**

4. **Foreskin** covering less than half the glans

5. Absolute **medical indication for circumcision** (balanitis or phimosis)

6. **Bleeding disorder** diagnosed by a doctor (eg, factor deficiency, coagulopathy, or platelet disorder requiring special precautions). [This exclusion also applies to therapeutic anticoagulation that results in a prolonged PT/INR or PTT.]

7. **HIV vaccine(s)** received in a prior HIV vaccine trial. For potential participants who received control/placebo in an HIV vaccine trial, the HVTN 914 PSRT will determine eligibility on a case-by-case basis

8. **Untreated clinical signs or symptoms of genitourinary or colonic infection**

9. **Any medical condition contraindicating circumcision or flexible sigmoidoscopy with biopsies**

10. **History of transactional sex** (ie, exchange of sex for money, shelter, food, or drugs) in the preceding 6 months

11. **Any medical, psychiatric, occupational, or other condition** that, in the judgment of the investigator, would interfere with, or serve as a contraindication to, protocol adherence, assessment of safety, or a participant's ability to give informed consent

12. **History of keloid scarring**

7.3 Participant termination from the study

Under certain circumstances, an individual participant may be terminated from participation in this study. Specific events that will result in early termination include:

- Participant refuses further participation,
- Participant relocates and remote follow-up is not possible,
- HVTN CRS determines that the participant is lost to follow-up,
- Participant becomes HIV-infected, or
- Investigator decides, in consultation with Protocol Team leadership, to terminate participation (eg, if participant exhibits inappropriate behavior toward clinic staff).

8 Clinical procedures

The schedule of clinical procedures is shown in Appendix G.

8.1 Informed consent

Informed consent is the process of ensuring that participants fully understand what will and may happen to them while participating in a research study. The informed consent form documents that a participant (1) has been informed about the potential risks, benefits, and alternatives to participation, and (2) is willing to participate in the study. Informed consent encompasses all written or verbal study information HVTN CRS staff provide to the participant, before and during the trial. HVTN CRS staff will obtain informed consent of participants according to HVTN policies and procedures.

The informed consent process continues throughout the study. Key study concepts should be reviewed periodically with the participant and the review should be documented. At each study visit, HVTN CRS staff should consider reviewing the procedures and requirements for that visit and for the remaining visits. Additionally, if any new information is learned that might affect the participants' decisions to stay in the study, this information will be shared with study participants. If necessary, participants will be asked to sign revised informed consent forms.

An HVTN CRS may employ recruitment efforts prior to the participant consenting. Participants must sign a screening or protocol-specific consent before any procedures are performed to determine eligibility. HVTN CRSs must submit recruitment and prescreening materials to IRBs/IECs for human subjects protection review and approval.

8.1.1 Informed consent form

The consent form describes all aspects of protocol participation, including screening and enrollment procedures. The study CRS is responsible for developing a consent form for local use, based on the sample protocol-specific consent form in Appendix A. This consent form must be developed in accordance with local IRB/IEC requirements and the principles of informed consent as described in Title 45, Code of Federal Regulations (CFR) Part 46 and Title 21 CFR, Part 50, and in the International Conference on Harmonisation (ICH) E6, Good Clinical Practice: Consolidated Guidance 4.8.

The study site is strongly encouraged to have its CAB review the local consent form. This review should include, but should not be limited to, issues of cultural competence, local language considerations, and the level of understandability.

Prior to implementing version 1 of this protocol, each site must have the protocol and site-specific protocol consent form(s) approved by its IRB/EC and any other applicable Regulatory Entity (RE). Prior to site activation, site-specific informed consent forms will be reviewed and approved by the DAIDS Protocol Registration Office (PRO) and/or HVTN Regulatory Affairs.

Regarding protocol registration, sites should follow procedures outlined in the current version of the DAIDS Protocol Registration Manual.

8.1.2 Assessment of Understanding

Study staff should ensure that participants fully understand the study before enrolling them. This process involves reviewing the informed consent form with the participant, allowing time for the participant to reflect on the procedures and issues presented, and answering all questions completely.

An Assessment of Understanding is used to document the participant's understanding of key concepts in this study. The participant must complete the Assessment of Understanding before enrollment. Staff may provide assistance in reading and understanding the questions and responses, if necessary. Participants must verbalize understanding of all questions answered incorrectly. This process and the participant's understanding of the key concepts should be recorded in source documentation at the site.

IRBs/IECs may require that a participant has signed either a screening or protocol-specific consent document prior to administering the Assessment of Understanding. The consent process (including the use of the Assessment of Understanding) should be explained thoroughly to the IRB/IEC, whose recommendations should be followed.

8.2 Pre-enrollment procedures

Screening procedures are done to determine eligibility and to provide a baseline for comparison of safety data. Baseline data are obtained during screening. Screening may occur over the course of several contacts/visits, up to and including before collection of all specimens required at day 0. All inclusion and exclusion criteria must be assessed within 56 days before enrollment, unless otherwise specified in the eligibility criteria (or below in this section).

After the appropriate informed consent has been obtained and before enrollment, the following procedures are performed:

- Medical history, documented in the case history record;
- Complete physical examination, including height, weight, vital signs and clinical assessments of: head, ears, eyes, nose, and throat; neck; lymph nodes; heart; chest; abdomen; extremities; neurological function; and skin;
- Assessment of STI signs and symptoms, including clinical examination for penile, anal, and perianal pathology;
- Assessment of concomitant medications the volunteer is taking, including prescription and nonprescription drugs, vitamins, topical products, alternative/complementary medicines (eg, herbal and health food supplements), recreational drugs, vaccinations, and allergy shots (record the complete generic name for all medications);
- Pre- and post-HIV test counseling, including HIV risk reduction counseling, performed in compliance with Peruvian guidelines for HIV counseling, testing, and referral (see section 8.4);

- Laboratory tests, including:
    - HIV test,
    - HSV-2 test (exempt from 56-day screening window),
    - Complete blood count (CBC)/differential/platelets, and
    - PT/PTT and INR;
- Risk reduction counseling (as described in section 8.4);
- Eligibility questionnaire, including assessment of sexual role preference; and
- Obtaining of volunteer demographics in compliance with the NIH Policy on Reporting Race and Ethnicity Data: Subjects in Clinical Research, Aug. 8, 2001 (available at http://grants.nih.gov/grants/guide/notice-files/NOT-OD-01-053.html).

8.3 Enrollment and follow-up visits

Enrollment is defined as collection of all specimens required at day 0. The time interval between screening and enrollment should not exceed 56 days.

The following procedures are performed at the enrollment visit and all scheduled follow-up visits:

- Risk reduction counseling (as described in section 8.4);
- Counseling on procedure-specific safety criteria (see sections 8.6.1 and 8.7.1);
- Assessment of new or unresolved social impacts (site staff will ask participant about the status of any unresolved social impacts and if he has experienced any new social impacts as a result of study participation);
- Assessment of new or continuing concomitant medications (as described in section 8.2);
- CASI questionnaire, including behavioral risk, personal **a**nd social impact assessment;
- Clinical laboratory tests, including CBC/differential/platelets; and
- Specimen collection (see Appendix F and Appendix G).

Additional procedures will be performed at scheduled visits as specified in Appendix G:

- Abbreviated physical examination, including weight, vital signs, and a symptom-directed evaluation by history and/or appropriate physical exam based on participant self-reported symptoms or complaints;

- Assessment of signs and symptoms of STIs, including clinical examination for penile, anal, and perianal pathology;
- Clinical laboratory tests including:
  - HIV test,
  - syphilis test,
  - HSV-2, and
  - *Neisseria gonorrhea* (NG) and *Chlamydia trachomatis* (CT) diagnostic testing in urine and rectal swabs;
- HIV infection assessment including pre-test counseling. A subsequent follow-up contact is conducted to provide post-test counseling and to report results to participant;
- Circumcision (includes pre- and post-procedure visit with provider performing the procedure);
- Flexible sigmoidoscopy with rectosigmoid biopsies (includes pre- and post-procedure visit with provider performing the procedure);
- Assessment of new or unresolved procedure-related events; and
- Evaluation to confirm proper healing after circumcision or sigmoidoscopy procedures, provide medical clearance to resume previously restricted sexual activity, and document any complications.

8.4 HIV counseling and testing

HIV testing will be conducted under the guidance of the HVTN Laboratory Program.

HIV counseling will be performed in compliance with Peruvian guidelines for HIV counseling and referral, which reflect current US Centers for Disease Control (CDC) counseling guidelines. Participants will be counseled at all scheduled visits during the trial on the avoidance of HIV infection.

In particular, counseling will focus on abstinence from insertive sex behavior during the six weeks following circumcision in order to decrease the risk of HIV acquisition prior to wound healing and also to reduce risk when opting for receptive anal sex during the same period. Counseling will also emphasize avoidance of receptive anal sex from 3 days before through 7 days after each sigmoidoscopy procedure.

Potential participants identified as being HIV-infected during screening are not enrolled. All participants who become HIV-infected during the study will be terminated from this study. Potential and enrolled participants identified as HIV-infected will be referred for medical treatment, counseling, and management of the HIV infection. These individuals may also be referred to appropriate ongoing clinical trials or observational studies.

8.5 STI assessment and treatment

In addition to clinical laboratory testing, as described in Appendix F, participants will be assessed for signs and symptoms of STIs at selected physical exams, described in Appendix G. The standard of care in Peru includes syndromic treatment of STIs. Participants exhibiting any signs or symptoms of an STI and/or testing positive by clinical laboratory test will receive treatment according to the Peruvian guidelines through the CRS.

8.6 Flexible sigmoidoscopy

Flexible sigmoidoscopy will be conducted at week 2 and week 27 to obtain rectosigmoid mucosal specimens for evaluation of mucosal anatomy, HIV target cells, innate resistance factors, and antigen-specific responses. Per individual, up to 25 sigmoid biopsy samples will be taken and up to 4 rectal biopsy samples will be taken. Fewer samples may be taken based on the judgment of the performing clinician.

8.6.1 Sigmoidoscopy biopsy-specific safety criteria

Rectosigmoid biopsies should not be performed if the participant meets any of the following criteria:

- Abnormality of the colorectal mucosa, which in the opinion of the clinician represents a contraindication to biopsy (including but not limited to presence of any unresolved injury, infectious or inflammatory condition of the local mucosa, or presence of hemorrhoids);
- Use of medications interfering with normal coagulation (eg, coumadin and heparin derivatives);
- Use of aspirin or other analgesic drug with anticoagulant properties during the preceding 7 days;
- Receptive anal intercourse or insertion of foreign object into the anus during the preceding 3 days;
- Refusal to agree to abstain from receptive anal intercourse and/or insertion of foreign objects into rectum for 7 days after biopsy;
- Any colorectal procedure within the prior 2 weeks, or
- Other condition, which, in the opinion of the clinician or site investigator, represents a contraindication to biopsy.

If the disqualifying condition resolves and the procedure can be rescheduled and performed within the specified visit window (see HVTN 914 *Study Specific Procedures*), then the biopsies may be performed.

8.7 Circumcision

Circumcision will be conducted during week 4 to obtain foreskin samples for evaluation of mucosal anatomy, HIV target cells, innate resistance factors, and antigen specific responses.

8.7.1 Circumcision-specific safety criteria

Circumcision should not be performed if the participant meets any of the following criteria:

- Abnormality of the penis, which in the opinion of the clinician represents a contraindication to circumcision (including but not limited to presence of any local unresolved injury, or infectious or inflammatory condition);
- Use of medications interfering with normal coagulation (eg, coumadin and heparin derivatives);
- Use of aspirin or other analgesic drug with anticoagulant properties during the preceding 7 days;
- Refusal to agree to abstain from insertive intercourse for 6 weeks post-circumcision or until clinician clearance, or
- Other condition, which, in the opinion of the clinician or site investigator, represents a contraindication to circumcision.

If the disqualifying condition resolves and the procedure can be rescheduled and performed within the specified visit window (see HVTN 914 *Study Specific Procedures*), then the circumcision may be performed.

8.8 Visit windows and missed visits

Visit windows are defined in HVTN 914 *Study Specific Procedures*. For a visit not performed within the window period, a Missed Visit form is completed. If the missed visit is one that required safety assessments or local safety labs, CRS staff should attempt to bring the participant in for an interim visit as soon as possible.

Procedures performed at an interim visit are limited to procedure-related event, safety and social impact assessments (including local safety labs and HIV testing), and CASI questionnaire.

8.9 Early termination visit

In the event of early participant termination, site staff should consider if the following assessments are appropriate: a physical examination, clinical laboratory tests (including CBC with differential, platelet count, STI testing, and HIV test), CASI questionnaire, and social impact assessment.

9 Laboratory

9.1 HVTN CRS laboratory procedures

The *HVTN Site Lab Reference Manual* provides further guidelines for operational issues concerning the clinical and processing laboratories. The manual includes guidelines for general specimen collection, special considerations for phlebotomy, specimen labeling, whole blood processing, HIV screening/diagnostic testing, and general screening and safety testing.

Tube types for blood collection are specified in Appendix F. For tests performed locally, the local lab may assign appropriate tube types.

In specific situations, the blood collection tubes will be redirected to another laboratory or will require study-specific processing techniques. In these cases, laboratory special instructions will be posted on the protocol-specific section of the HVTN website.

9.2 Total blood volume

Required blood volumes per visit are shown in Appendix F. Not shown is any additional blood volume that would be required if a safety lab needs to be repeated (ie, beyond the 15 milliliters [mL] reserved for that purpose). Circumcision procedures are associated with blood loss of 10-30 mL; rectosigmoid tissue biopsies via flexible sigmoidoscopy are associated with blood loss of 5-20 mL. The total blood volume drawn for each participant will not exceed 500 mL in any 56-day (8-week) period, including allowances for blood loss during and after surgical procedures.

9.3 Mucosal specimen processing and preparation

Rectosigmoid biopsies and foreskin will be processed for real-time (RT)-PCR analysis, histology, and flow cytometry, with the foreskin undergoing additional processing for explant cultures. All specimen processing and preparation including leukocyte extraction, stimulation, flow cytometry staining, and explant cultures will be carried out in Lima whereas the assays themselves will be performed in the U.S. Information regarding adherence to specimen processing and preparation will be collected for the purpose of recording protocol deviations.

9.4 Immunological assays

9.4.1 Antibody-based detection

Cytokines and chemokines will be evaluated in plasma and supernatants of explant cultures with the Luminex multiplex assay and/or enzyme-linked immunosorbent assay (ELISA). Markers of immune activation in HIV target cells will be examined in plasma specimens from weeks 2, 3, 4, 5, 10, 26, 27, and 28 and will be compared to baseline samples collected at weeks 0 and 1. For example, levels of CCR5 and CXCR4 binding chemokines, such as RANTES, MIP1α, MIP1β, and SDF-1 may be examined in plasma

and explant culture supernatants. Data will be reported as concentration of these markers at said timepoints.

9.4.2 Real time (RT)-PCR

RNA isolated from PBMCs, foreskin tissue, rectal biopsies, and sigmoid biopsies will be used to measure bulk levels of chemokine receptors CCR5 and CXCR4, and their ligands RANTES, MIP1α, MIP1β, and SDF-1 *in situ*. Other markers of cellular activation (eg, Ki67, TNF-α) and proteins promoting innate antiviral protection (eg, langerin, APOBEC3, and TRIM5α) may be analyzed.

9.4.3 Flow cytometry

Fresh and frozen PBMCs, cells from the cultured explants of the foreskin, and isolated leukocytes from the rectosigmoid colon and foreskin will be assessed by flow cytometry to quantify target cells of HIV infection (T cells, dendritic cells, and macrophages). Markers for quantifying target cells of HIV infection may include CD3, CD4, CD8, CD163, CD14, CD1c, and/orCD1a. Also measured will be markers of activation, innate resistance, and/or chemokine receptors on HIV target cells. Furthermore, ICS staining of PBMCs and of isolated leukocytes from the foreskin and rectosigmoid colon will be conducted to measure antigen-specific responses (eg, TNF-α, IFN-γ, IL-2, perforin, and/or granzyme B) to Staphylococcal enterotoxin B and adenovirus as sample antigens. Other cellular markers and viral antigens may also be examined.

9.4.4 Histopathology and immunofluorescence microscopy

Formalin-fixed and paraffin-embedded foreskin, sigmoid, and rectal tissue samples will be stained for imunofluorescence microscopy to analyze the keratin layer thickness and the localization of HIV target cells within the epidermis and dermis. Paraffin-embedded samples will also be used to characterize CD4+ T cells in tissue, which will be tested for correlation with the flow cytometry approach. CD4+ and CD8+ T cells will be measured in addition to CCR5 levels, Ki67 levels, and their proximity to the external surface of the mucosa.

Optimal cryofreezing temperature (OCT) media-embedded samples will be analyzed to characterize the myeloid HIV targets, and the density of HIV receptors present in the tissue (eg, DC Sign, CCR5, CD4 in myeloid cells). Staining for target cells expressing the factors that promote HIV integration and replication, such as pSTAT, pJUNK, and nuclear NFKB translocation, may also be done. Moreover, pSTAT3 staining may be conducted with the aim of identifying areas of epithelial damage in the mucosa.

9.5 Other use of stored specimens

The HVTN aims not only to test vaccine candidates but also to continue to explore the correlates of immunity to HIV. In order to do so, the HVTN intends to store blood, cell, and tissue samples from participants. These samples will be used for other testing and research related to furthering the understanding of HIV or vaccines to the extent authorized in each study site’s informed consent form, or as otherwise authorized under applicable law. Other testing on specimens will only occur, at a minimum, after review and approval by the HVTN and the IRB of the researcher requesting the specimens.

The protocol sample informed consent form is written so that the participant either explicitly allows or does not allow sample storage for other research when he or she signs the form. Participants who initially agree to other use of their samples may rescind their approval once they enter the study; such participants will still remain in this study. If a participant decides against allowing other research using his or her samples, or at any time rescinds prior approval for such other use, the study site investigator or designee must notify HVTN Regulatory Affairs in writing. In either case, after study analyses are complete, the HVTN Laboratory Program will request that the repository destroy all specimens with the participant identification numbers (PTIDs) of all participants who do not agree to other use of their samples. HVTN Core will report the destruction of relevant specimens to the participants' site Principal Investigators (PIs).

Study sites must notify HVTN Regulatory Affairs if institutional or local governmental requirements pose a conflict with or impose restrictions on the use of stored specimens.

9.6 Biohazard containment

As the transmission of HIV and other blood-borne pathogens can occur through contact with contaminated needles, blood, and blood products, appropriate precautions will be employed by all personnel in the drawing of blood and shipping and handling of all specimens for this study, as currently recommended by the CDC and the NIH or other locally appropriate agencies.

All dangerous goods materials, including Biological Substances, Category A or Category B, must be transported according to instructions detailed in the International Air Transport Association Dangerous Goods Regulations.

10 Safety oversight and safety review

As no investigational product will be administered in this study, specific tests for clinical monitoring, monitoring for adverse events (AEs) or adverse events requiring expedited reporting to DAIDS (EAE), AE grading, and reporting will not be performed. Participants will be monitored for procedure-related safety events and these events will be reported regularly to the PSRT. The PSRT will review these reports and may make recommendations concerning participants' continuation in the study.

10.1 HVTN 914 PSRT

The PSRT is composed of the following members:

- DAIDS medical officer representative,
- Protocol chair and cochair,
- Protocol Team leader,
- Core medical monitor, and
- SDMC Clinical Affairs safety associate.

The clinician members of the PSRT are responsible for decisions related to participant safety.

The Protocol Team clinic coordinator, project manager, clinical trial manager, and others may also be included in PSRT meetings.

10.2 SDMC roles and responsibilities in safety monitoring

The roles and responsibilities of the SDMC in relation to safety monitoring include:

- Maintaining a central database management system for HVTN clinical data;
- Providing reports of clinical data to appropriate groups such as the HVTN 914 PSRT (see section 10.1);
- Daily monitoring for clinical data that meet criteria for prompt PSRT notification (see section 10.3.2);
- Querying HVTN CRSs for additional information regarding reported clinical data; and
- Providing support to the HVTN 914 PSRT.

10.3 Safety reporting

10.3.1 Submission of safety forms to SDMC

Sites must submit all safety forms (eg, procedure-related events, local lab results, concomitant medications) before the end of the next business day after receiving the information. The forms should not be held in anticipation of additional information at a later date unless otherwise instructed by the SDMC. If additional information is received at a later date, the forms should be updated and refaxed before the end of the next business day after receiving the new information.

10.3.2 Procedure-related event reporting

Any of the following events should be reported to SDMC Clinical Affairs on the appropriate case report form (CRF):

- Rectal or other procedure-site hemorrhage or hematoma,
- Bowel perforation or other anatomic injury,
- Locoregional pain,
- Local infection,
- Local edema,
- Adverse drug reaction, or
- Other procedure-related events.

Procedure-related events assessed by the CRS as grade 3 or higher should be reported immediately to SDMC Clinical Affairs. Upon receipt of information concerning any Grade 3 or higher event, the PSRT will conduct a prompt review of the participant's clinical data and determine if the participant may continue in further study-related procedures. Prompt PSRT review is defined as review within 48 hours of SDMC notification (excluding weekends, US federal holiday, and Peruvian national holidays). In addition, the PSRT may determine if the study can continue with or without amendment.

The maximum level of severity of the 7-day cumulative procedure-related event findings are reported for each participant. When determining the severity grade for reported symptoms, sites will use the DAIDS Table for Grading the Severity of Adult and Pediatric Adverse Events, (Publish Date: December, 2004; Clarification: August 2009). The DAIDS AE Grading Table is available on the RSC web site at http://rsc.tech-res.com/safetyandpharmacovigilance/.

Procedure-related events that are not specifically identified in the DAIDS AE grading table are reported using the category "Estimating Severity Grade" located on the first page of the DAIDS AE grading table.

10.4 Procedure-specific safety criteria assessment

Prior to conducting a procedure, the site is responsible for ensuring that a participant meets all procedure-specific safety criteria (see sections 8.6.1 and 8.7.1). If a condition is noted during this assessment that, in the opinion of the performing clinician, represents a contraindication to sampling, the procedure should be postponed or cancelled.

10.5 Study termination

This study may be terminated early by the determination of the HVTN 914 PSRT, NIH, or Office for Human Research Protections (OHRP). In addition, the conduct of this study may be terminated by the determination of the local IRB or IEC, or of the appropriate local or national regulatory authority.

11 Protocol conduct

Except as specified within the protocol, this study will be conducted in compliance with the principles of GCP and according to standard DAIDS and HVTN policies and procedures, including procedures for the following:

- Protocol registration, activation, and implementation;
- Informed consent, screening, and enrollment;
- Clinical and safety assessments;
- Safety monitoring and reporting;
- Data collection and documentation;
- Study follow-up and close-out;
- Quality control;
- Protocol monitoring and compliance;
- Risk reduction counseling; and
- Specimen collection, processing, and analysis.

Any other policies or procedures that vary from DAIDS and HVTN standards or require additional instructions will be described in the HVTN 914 *Study Specific Procedures*.

11.1 Overview of data collection methods

Clinical research data will be collected in a secure electronic data management system by the assigned SDMC. Data will be extracted and provided to the protocol statistician for statistical analysis.

11.1.1 Source documents and data entry at sites

Standard GCP will be followed to ensure accurate, reliable, and consistent data collection. Each participating site will maintain appropriate medical and research records for this trial, in compliance with ICH, GCP, regulatory, network, and institutional requirements for the protection of confidentiality of participants.

HVTN sites will follow the *DAIDS Standard Operating Procedure on Source Documentation*, Version 2 or any later version in managing source documentation for the trial. Source document information may include but is not limited to:

- Signed informed consent documents;
- Dates of visits;

- Documentation of the study eligibility evaluation;
- Reported laboratory results;
- Procedure-related event evaluations; and
- Participant-reported concomitant medications.

CRFs and laboratory reports will be reviewed by the site clinical team responsible for ensuring that they are accurate and complete. Many HVTN CRFs are designed to be used as source documents. HVTN CRSs complete a source documentation table to indicate which CRFs the site will use as source documents for the trial.

11.1.2 Participant confidentiality

Documentation, data, and all other information generated for a participant will be held in strict confidence. No identifying participant information concerning the study or the data will be released to any unauthorized third party without prior written approval of the participant except as necessary for monitoring by the IRB/IEC, the study sponsor, and the OHRP. Information about a study participant also may be released when required by law. Participants must be made aware in the informed consent document of the occasions when information may be released without their consent. In addition, if information is released, either by accident or deliberately without a participant's consent, the site must attempt to notify the participant of the release, complete a Protocol Event Form (see section 11.2.2), and notify their IRB/IEC.

The study database assembled by the SDMC will identify study participants only by a study identification number and will not contain identifying information such as name, address, national identification number, medical record number, or personal contact information.

11.1.3 Lab data transfer

Data generated at central and regional laboratories will be transferred directly from the laboratory to the SDMC by secure means and with procedures that ensure the integrity of the data.

11.1.4 Storage of source documents and completed CRFs

All study data must be verifiable to the source documentation. A file containing all the source documents will be maintained for each study participant at the study site. Source documentation will be available for review to ensure that the collected data are consistent with the CRFs.

CRFs, source documents, and other supporting documents will be kept in a secure location.

11.2 HVTN CRS monitoring

To ensure protection of study participants, compliance with the protocol, and accuracy and completeness of records, site monitors under contract to NIAID may visit participating CRSs to review the individual subject records, including consent forms, CRFs, supporting data, laboratory specimen records, and medical records (physicians' progress notes, nurses' notes, and individuals' hospital charts). The monitors will inspect sites' regulatory files to ensure that regulatory requirements are being followed.

11.2.1 Access to source documents

Because this study is sponsored by NIAID, each site must permit authorized representatives of NIAID and regulatory agencies to examine (and, when required by applicable law, to copy) clinical records for the purposes of quality assurance reviews, audits, and evaluation of the study's safety and progress.

Additionally, each site must permit representatives of the HVTN, SDMC, and related contractors to examine clinical records for the purposes of quality assurance reviews, audits, and evaluation of the study's safety and progress.

11.2.2 Protocol events

A protocol event is defined as an individual incident or omission in study conduct that results in significant added risk to the participant, or nonadherence to significant protocol requirements, or nonadherence to the *International Conference on Harmonisation E6: Guideline for Good Clinical Practice*.

The nonadherence may be on the part of either a participant, the investigator, or the study site staff.

It is the responsibility of the site to identify and report protocol events according to the guidelines of the sponsor and the local IRB/IEC per their guidelines. The site must also report protocol events to the HVTN using the Protocol Event Form. The site PI and study staff are responsible for knowing and adhering to their IRB requirements.

In response to noted protocol events, site personnel are to implement corrective actions promptly, as necessary.

11.3 Social impacts

Participants in this study risk experiencing discrimination or other personal problems as a result of being in the study. The HVTN CRS is obliged to provide advocacy for and assistance to participants regarding negative social impacts associated with the study. If HVTN CRS staff have questions regarding how to assist a participant dealing with a social impact, a designated NIAID representative can be contacted.

11.4 Study participant reimbursement

Reimbursement of study participants for attendance at study visits is at the discretion of each study site. Reimbursement should be comparable to the reimbursement offered for similar research in the local community, if possible. The study site is encouraged to confer with its local CAB in deciding appropriate reimbursement.

The study consent submitted to the site IRB/IEC will state the plan for reimbursement (if any). The HVTN relies upon local IRBs/IECs to determine whether the proposed plan for reimbursement meets ethical requirements in the local context. The exact amounts may be modified during the course of the study in consideration of changes in costs such as bus fares, exchange rates, childcare, or other factors that affect the ability of a participant to comply with study visit requirements. Reviewing IRBs/IECs must be made aware of the changes in reimbursement before they occur. Study participants will not be charged for study procedures, research clinic visits, research-related examinations, or research-related laboratory tests.

The HVTN does not allow reimbursement that induces a study participant to remain in the study against his or her will. A lump sum reimbursement at trial completion solely to encourage retention is unacceptable.

12 Version history

The Protocol Team may modify the original version of the protocol. Modifications are made to HVTN protocols via clarification memos, letters of amendment, or full protocol amendments.

The table below describes the version history of, and modifications to, Protocol HVTN 914.

Protocol history and modifications

Date	Protocol version	Protocol modification	Comment
August 16, 2010	Version 1	Original protocol	

13 Document references (other than literature citations)

Other documents referred to in this protocol, and containing information relevant to the conduct of this study, include:

Assessment of Understanding. Accessible through the HVTN protocol-specific website.

Current CDC Guidelines. Revised Recommendations for HIV Testing of Adults, Adolescents, and Pregnant Women in Health-Care Settings. Available at http://www.cdc.gov/mmwr/PDF/rr/rr5514.pdf.

Division of AIDS (DAIDS) Clinical Research Policies and Standard Procedures Documents. Available at http://www3.niaid.nih.gov/research/resources/DAIDSClinRsrch/

Division of AIDS Protocol Registration Manual. Available at http://www.niaid.nih.gov/LabsAndResources/resources/DAIDSClinRsrch/Documents/prmanual.pdf

Division of AIDS Table for Grading the Severity of Adult and Pediatric Adverse Events. Version 1.0, December 2004 (Clarification August 2009). Available at http://rsc.tech-res.com/safetyandpharmacovigilance/

HVTN 914 Special Instructions. Accessible through the HVTN protocol-specific website.

HVTN 914 Study Specific Procedures. Accessible through the HVTN protocol-specific website.

HVTN Site Lab Reference Manual. Accessible through the HVTN website.

HVTN Manual of Operations. Accessible through the HVTN website.

International Conference on Harmonisation (ICH) E6 (R1), *Guideline for Good Clinical Practice*: section 4.8, *Informed consent of trial subjects*. Available at http://www.emea.europa.eu/pdfs/human/ich/013595en.pdf.

Participants' Bill of Rights and Responsibilities. Accessible through the HVTN website.

NIH Policy on Reporting Race and Ethnicity Data: Subjects in Clinical Research. Available at http://grants1.nih.gov/grants/guide/notice-files/NOT-OD-01-053.html.

Requirements for Source Documentation in DAIDS Funded and/or Sponsored Clinical Trials. Available at http://www3.niaid.nih.gov/research/resources/DAIDSClinRsrch/ClinicalSite.htm

Title 21, Code of Federal Regulations, Part 50. Available at http://www.access.gpo.gov/nara/cfr/waisidx_08/21cfrv1_08.html.

Title 45, Code of Federal Regulations, Part 46. Available at http://www.access.gpo.gov/nara/cfr/waisidx_07/45cfrv1_07.html.

See section 15 for literature cited in the background and statistics sections of this protocol.

14 Acronyms and abbreviations

Ad5	adenovirus type 5
AE	adverse event
ART	antiretroviral therapy
CAB	Community Advisory Board
CASI	computer-assisted self interview
CBC	complete blood count
CDC	US Centers for Disease Control and Prevention
CFR	Code of Federal Regulations
CIOMS	Council for International Organizations of Medical Sciences
CRF	case report form
CRS*	clinical research site
CT	*Chlamydia trachomatis*
DAIDS	Division of AIDS (US NIH)
FHCRC	Fred Hutchinson Cancer Research Center
GCP	Good Clinical Practice
HIV	human immunodeficiency virus
HSV-2	herpes simplex virus type 2
HVTN	HIV Vaccine Trials Network
ICH	International Conference on Harmonisation
ICS	intracellular cytokine staining
IEC	Independent Ethics Committee
IFN-γ	interferon gamma
IMPACTA	Asociación Civil Impacta Salud y Educación (Lima, Peru)
INR	international normalized ratio
IRB	Institutional Review Board
LC	Langerhans cell
MHA-TP	microhemagglutination for *Treponema pallidum*
mL	milliliter
MSM	men who have sex with men
MTF	male-to-female (transgender)
NG	*Neisseria gonorrhea*
NIAID	National Institute of Allergy and Infectious Diseases (US NIH)
NIH	US National Institutes of Health
NMRCD	(US) Naval Medical Research Center Detachment
OHRP	US Office for Human Research Protections
PBMC	peripheral blood mononuclear cell
PCR	polymerase chain reaction
PI	Principal Investigator
PSRT	Protocol Safety Review Team

PT	prothrombin time
PTT	partial thromboplastin time
RAB	DAIDS Regulatory Affairs Branch
RSC	DAIDS Regulatory Support Center
RNA	ribonucleic acid
RPR	rapid plasma reagin
SAE	serious adverse event
SCHARP	Statistical Center for HIV/AIDS Research and Prevention
SDMC	statistical and data management center
STI	sexually transmitted infection

[*] CRSs were formerly referred to as HIV Vaccine Trial Units (HVTUs). Conversion to use of the term CRS is in process, and some HVTN documents may still refer to HVTUs.

15 Literature cited

1. Council for International Organizations of Medical Sciences (CIOMS). International ethical guidelines for biomedical research involving human subjects. Bull Med Ethics **2002**;17-23.

2. UNAIDS. Ethical considerations in HIV preventive vaccine research. **2000**;UNAIDS/04.07E.

3. The National Commission for the Protection of Human Subjects of Biomedical and Behavioral Research. The Belmont Report: Ethical Principles and Guidelines for the Protection of Human Subjects of Research. **1979**.

4. Auvert B, Taljaard D, Lagarde E, Sobngwi-Tambekou J, Sitta R, Puren A. Randomized, controlled intervention trial of male circumcision for reduction of HIV infection risk: the ANRS 1265 Trial. PLoS Med **2005**;2:e298.

5. Bailey RC, Moses S, Parker CB, Agot K, Maclean I, Krieger JN, Williams CF, Campbell RT, Ndinya-Achola JO. Male circumcision for HIV prevention in young men in Kisumu, Kenya: a randomised controlled trial. Lancet **2007**;369:643-56.

6. Gray RH, Kigozi G, Serwadda D, Makumbi F, Watya S, Nalugoda F, Kiwanuka N, Moulton LH, Chaudhary MA, Chen MZ, Sewankambo NK, Wabwire-Mangen F, Bacon MC, Williams CF, Opendi P, Reynolds SJ, Laeyendecker O, Quinn TC, Wawer MJ. Male circumcision for HIV prevention in men in Rakai, Uganda: a randomised trial. Lancet **2007**;369:657-66.

7. Patterson BK, Landay A, Siegel JN, Flener Z, Pessis D, Chaviano A, Bailey RC. Susceptibility to human immunodeficiency virus-1 infection of human foreskin and cervical tissue grown in explant culture. Am J Pathol **2002**;161:867-73.

8. Dinh MH, McRaven MD, Kelley Z, Penugonda S, Hope TJ. Keratinization of the adult male foreskin and implications for male circumcision. AIDS **2010**.

9. McCoombe SG, Short RV. Potential HIV-1 target cells in the human penis. AIDS **2006**;20:1491-5.

10. Jameson DR, Celum CL, Manhart L, Menza TW, Golden MR. The Association Between Lack of Circumcision and HIV, HSV-2, and Other Sexually Transmitted Infections Among Men Who Have Sex With Men. Sex Transm Dis **2010**;37:147-52.

11. Vittinghoff E, Douglas J, Judson F, McKirnan D, MacQueen K, Buchbinder SP. Per-contact risk of human immunodeficiency virus transmission between male sexual partners. Am J Epidemiol **1999**;150:306-11.

12. Buchbinder SP, Vittinghoff E, Heagerty PJ, Celum CL, Seage GR, III, Judson FN, McKirnan D, Mayer KH, Koblin BA. Sexual risk, nitrite inhalant use, and lack of circumcision associated with HIV seroconversion in men who have sex with men in the United States. J Acquir Immune Defic Syndr **2005**;39:82-9.

13. Bartholow BN, Goli V, Ackers M, McLellan E, Gurwith M, Durham M, Greenberg AE. Demographic and behavioral contextual risk groups among men who have sex with men participating in a phase 3 HIV vaccine efficacy trial: implications for HIV prevention and behavioral/biomedical intervention trials. J Acquir Immune Defic Syndr **2006**;43:594-602.

14. Millett GA, Flores SA, Marks G, Reed JB, Herbst JH. Circumcision status and risk of HIV and sexually transmitted infections among men who have sex with men: a meta-analysis. JAMA **2008**;300:1674-84.

15. Qin Q, Zheng XY, Wang YY, Shen HF, Sun F, Ding W. Langerhans' cell density and degree of keratinization in foreskins of Chinese preschool boys and adults. Int Urol Nephrol **2009**.

16. Donoval BA, Landay AL, Moses S, Agot K, Ndinya-Achola JO, Nyagaya EA, Maclean I, Bailey RC. HIV-1 target cells in foreskins of African men with varying histories of sexually transmitted infections. Am J Clin Pathol **2006**;125:386-91.

17. Buchbinder SP, Mehrotra DV, Duerr A, Fitzgerald DW, Mogg R, Li D, Gilbert PB, Lama JR, Marmor M, Del Rio C, McElrath MJ, Casimiro DR, Gottesdiener KM, Chodakewitz JA, Corey L, Robertson MN. Efficacy assessment of a cell-mediated immunity HIV-1 vaccine (the Step Study): a double-blind, randomised, placebo-controlled, test-of-concept trial. Lancet **2008**;372:1881-93.

18. Buchbinder S. Clinical follow-up in the Step Study. HVTN Full Goup Meeting, Washington, D.C.May 12, 2009, **2009**.

19. Guanira J. How willing are gay men to "cut off" the epidemic? Circumcision among MSM in the Andean Region. 4th International AIDS Society Conference on HIV Pathogenesis, Treatment and Prevention, Sydney, Australia, **2007**.

20. Guanira J, Lama J, Goicochea P, Segura P, Montoya O, Montano S, Kochel T, Grant R, Sanchez J. HIV incidence and syphilis rates among men who have sex with men at high risk for HIV-1 infection in 5 Andean cities. 14th

Conference on Retroviruses and Opportunistic Infections, Los Angeles, California, USA.February 15-28, 2007.Abstract no.961., **2007**.

21. Lama JR, Lucchetti A, Suarez L, Laguna-Torres VA, Guanira JV, Pun M, Montano SM, Celum CL, Carr JK, Sanchez J, Bautista CT, Sanchez JL. Association of herpes simplex virus type 2 infection and syphilis with human immunodeficiency virus infection among men who have sex with men in Peru. J Infect Dis **2006**;194:1459-66.

22. Clark JL, Espinosa B, Leon SR, Hall ER, Salvatierra HJ, Caceres CF, Klausner JD, Coates TJ. Absence of lymphogranuloma venereum infection among high-risk men who have sex with men in Lima, Peru. Int J STD AIDS **2008**;19:427-8.

23. Celum C, Wald A, Hughes J, Sanchez J, reid S, Delany-Moretlwe S, Cowan F, Casapia M, Ortiz A, Fuchs J, Buchbinder S, Koblin B, Zwerski S, Rose S, Wang J, Corey L. Effect of aciclovir on HIV-1 acquisition in herpes simplex virus 2 seropositive women and men who have sex with men: a randomised, double-blind, placebo-controlled trial. Lancet **2008**;371:2109-19.

24. Zhu J, Hladik F, Woodward A, Klock A, Peng T, Johnston C, Remington M, Magaret A, Koelle DM, Wald A, Corey L. Persistence of HIV-1 receptor-positive cells after HSV-2 reactivation is a potential mechanism for increased HIV-1 acquisition. Nat Med **2009**;15:886-92.

25. Krieger JN, Mehta SD, Bailey RC, Agot K, Ndinya-Achola JO, Parker C, Moses S. Adult male circumcision: effects on sexual function and sexual satisfaction in Kisumu, Kenya. J Sex Med **2008**;5:2610-22.

Appendix B: Table of procedures (for sample informed consent form)

	Screening	Weeks after enrollment visit									
		W0	W1	W2	W3	W4	W5	W10	W26	W27	W28
Medical history	√										
Complete physical	√										
Brief physical		√		√	√	√	√	√	√	√	√
HIV testing/counseling	√								√		
Interview/questionnaire	√	√			√	√	√	√	√	√	√
Computer questionnaire		√	√	√	√	√	√	√	√	√	√
Risk reduction counseling	√	√	√	√	√	√	√	√	√	√	√
Blood drawn	√	√	√	√	√	√	√	√	√	√	√
Urine test		√							√		
Rectal swabs		√							√		
Rectal/colon biopsy				√						√	
Circumcision						√					

Appendix C: Medication information sheet: Sigmoidoscopy

Before the sigmoidoscopy begins, you will be given a mixture of medications through an intravenous (IV) tube that will be inserted in your arm. This mixture may include **midazolam**, **pethidine**, and **hyoscine**. Midazolam is used to reduce anxiety. Pethidine is a used to relieve pain. Hyoscine is used to relieve intestinal spasms.

These medicines and similar medicines are used routinely around the world for sigmoidoscopies. Their high degree of effectiveness and their low rate of side effects is why they are being used for your sigmoidoscopy.

Some other medicines may interact with these medications. **Tell the doctor about any medicines you are taking.**

During any sigmoidoscopy, your medical condition will be monitored closely. The doctor will provide any needed medical attention. During the first 24 hours after the procedure, you might notice some side effects. **The most common reported side effects are**: constipation, lightheadedness, loss of appetite, blurred vision; changes in blood pressure, breathing, and heartbeats; coughing; dizziness; drowsiness; dry mouth; headache; hiccups; short-term memory loss; slurred speech.

You should tell your doctor if any of these symptoms becomes bothersome or lasts beyond the 2nd or 3rd day after your procedure.

We do not expect any serious side effects to using these medications for your sigmoidoscopy. However, medications can have serious or even life threatening side effects. **Tell the doctor immediately if you experience any of the following**: allergic reactions (rash; hives; difficulty breathing; tightness in the chest; swelling of the mouth, face, lips, or tongue); chest pain; difficulty urinating; fainting; fast, slow, or irregular heartbeat; mental or mood changes; numbness of an arm or a leg; seizure; severe or persistent dizziness; sudden severe headache, nausea, vomiting, or stomach pain; tremor; vision changes.

Appendix D: Medication information sheet: Lidocaine

Before the circumcision begins, you may be given **lidocaine**, a local anesthetic (numbing medication). It works by blocking nerve signals in your body. Lidocaine is given as an injection near the body area to be numbed.

Lidocaine is generally considered safe and effective at keeping people from feeling pain. That is why it is being used for this procedure.

Lidocaine may interact with other medications. **Tell the doctor about any medicines you are taking.**

Tell the doctor if you have liver or kidney disease, heart disease, coronary artery disease, circulation problems, or a history of fevers. Tell the doctor if you are allergic to lidocaine or to any other type of numbing medicine.

During the circumcision, the doctor will monitor your medical condition. The doctor will provide any needed medical attention. You might notice some side effects. **The most common side effects reported for lidocaine are** mild dizziness; nausea or vomiting; and, numbness in places where the medicine is accidentally applied.

Because you may feel dizzy or have impaired judgment, you should not drive for the rest of the day after you receive lidocaine.

We do not expect any serious side effects to using lidocaine for your circumcision. However, medications can have serious or even life-threatening side effects. **Tell the doctor immediately if you experience any of the following**: feeling anxious, shaky, restless, or depressed; drowsiness; ringing in your ears; blurred vision; confusion; twitching or seizure (convulsions); fast heart rate; rapid breathing; feeling hot or cold; weak or shallow breathing, slow heart rate, weak pulse; feeling like you might pass out.

Appendix E: Medication information sheet: Midazolam

Before the circumcision, you may be offered **midazolam**. Midazolam is used to relieve anxiety. It is given by injection into a large muscle.

Midazolam is generally considered safe and effective at reducing anxiety. That is why it is being used for this procedure.

Midazolam may interact with other medications. **Tell the doctor about any medicines you are taking.**

These are the most common side effects when using midazolam: blurred vision; changes in blood pressure; breathing, and heartbeats; coughing; dizziness; drowsiness; dry mouth; headache; hiccups; nausea; short-term memory loss; slurred speech; vomiting.

We do not expect any serious side effects with using midazolam for your circumcision. However, medications can have serious, even life-threatening side effects. **Tell the doctor immediately if you experience of the following**: rash; hives; difficulty breathing; swelling of the mouth, face, lips, or tongue; agitation; chest pain or tightness; combativeness; pain, swelling, or redness at the injection site; slow or difficult breathing; unusual or involuntary muscle movements or muscle tremor.

Appendix F: Laboratory procedures

					Tube volume (mL)											
				Visit:	**1**	**2**	**3**	**4**	**5**	**6**	**7**	**8**	**9**	**10**	**11**	
				Day:		D0	D7	D14	D21	D28	D35	D70	D182	D189	D196	
				Week:		W0	W1	W2	W3	W4	W5	W10	W26	W27	W28	
**Description**	**Processing location**	**Intermediary processing/ storage location**	**Assay location**	**Tube**	Screening	Baseline 1	Baseline 2									**Total**
BLOOD COLLECTION																
Screening, diagnostic or safety assays																
HIV test	Local lab	Local lab	Local lab	SST	5	—	—	—	—	—	—	—	3	—	—	**8**
Syphilis	Local lab	Local lab	Local lab	SST	—	10	—	—	—	—	—	—	3	—	—	**13**
HSV-2	Local lab	Local lab	Local lab	SST	5	_	—	—	—	—	—	—	4	—	—	**9**
CBC/ Diff/ platelets	Local lab	Local lab	Local lab	EDTA	3	3	3	3	3	3	3	3	3	3	3	**33**
PT/PTT	Local lab	Local lab	Local lab	Citrate	4	—	—	—	—	—	—	—	—	—	—	**4**
Immunogenicity assays																
Flow cytometry and ICS (fresh PBMCs)	Local lab	NMRCD	FHCRC	ACD	—	—	—	20	—	20	—	—	—	20	—	**60**
Flow cytometry and ICS (frozen PBMCs)	Local lab	CSR	FHCRC	ACD	—	50	50	40	50	50	50	50	50	50	50	**490**
RT-PCR	Local lab	CSR	FHCRC	ACD	—	10	10	10	10	10	10	10	10	10	10	**100**
Luminex multiplex assay / ELISA	Local lab	CSR	FHCRC	ACD	—	z	z	z	z	z	z	z	z	z	z	**0**
**Visit Total**					**17**	**73**	**63**	**73**	**63**	**83**	**63**	**63**	**73**	**83**	**63**	**717**
**Maximum 56-Day total**					**17**	**90**	**153**	**246**	**309**	**422**	**485**	**395**	**73**	**176**	**239**	
URINE COLLECTION																
NG/CT	Local lab	Local lab	Local lab		—	X	—	—	—	—	—	—	X	—	—	
RECTAL SWAB																
NG/CT	Local lab	FHCRC	KCPHD		—	X	—	—	—	—	—	—	X	—	—	
CIRCUMCISION																
Foreskin	NMRCD	NMRCD	FHCRC		—	—	—	—	—	X	—	—	—	—	—	
RECTOSIGMOID BIOPSY																
Rectosigmoid tissue	NMRCD	NMRCD	FHCRC		—	—	—	X	—	—	—	—	—	X	—	

CSR = Central specimen repository
HVTN Laboratory Program includes FHCRC = Fred Hutchinson Cancer Research Center (Seattle, Washington, USA)
Non-HVTN laboratories: NMRCD = Naval Medical Research Center Detachment (Lima, Peru); KCPHD = King County Public Health Department (Seattle, Washington, USA)
Screening may occur over the course of several contacts/visits up to and including day 0 prior to collection of all specimens required at day 0.
Local labs may assign appropriate alternative tube types for locally performed tests.
HSV-2 and syphilis testing at week 26 will be conducted only on participants who test negative at Visit 2.
At Visit 1 (screening), blood for HIV and HSV-2 testing will be collected in a single 10mL SST tube. At Visit 2 (Day 0), blood for syphilis testing will be drawn using a single 10mL SST tube. At Visit 9 (D182), blood for HIV, syphilis, and HSV-2 testing will be collected in a single 10 mL SST tube.
z = 5mL of plasma will be taken from ACD tubes during PBMC processing at the site processing lab.

Two baseline collections (at Visits 2 and 3) will be done to examine intra-person difference.

Maximum 56-day total for visits 6, 7, and 8 include 50mL of maximum estimated blood loss resulting from rectosigmoid biopsies and circumcision.

Maximum 56-day total for visits 4, 5, 10, and 11 includes 20mL of maximum estimated blood loss resulting from rectosigmoid biopsy.

Appendix G: Procedures at CRS

Visit:	01[a]	02	03	04	05	06	07	08	09	10	11
Day:		D0	D7	D14	D21	D28	D35	D70	D182	D189	D196
Week:		W0	W1	W2	W3	W4	W5	W10	W26	W27	W28
Procedure	Scr.										
**Study procedures**											
Signed informed consent	X	—	—	—	—	—	—	—	—	—	—
Assessment of understanding	X	—	—	—	—	—	—	—	—	—	—
Medical history	X	—	—	—	—	—	—	—	—	—	—
Complete physical exam	X	—	—	—	—	—	—	—	—	—	—
Abbreviated physical exam	—	—	X	—	X	X	X	X	X	X	X
Assessment of STI signs and symptoms	X	—	X	—	X	—	X	X	X	—	X
Evaluation to confirm proper healing	—	—	—	—	X	—	—	X	—	—	X
Pre- and post-HIV test counseling	X	—	—	—	—	—	—	—	X	—	—
Risk reduction counseling	X	X	X	X	X	X	X	X	X	X	X
Counseling on procedure-specific safety criteria	X	X	X	X	X	X	X	X	X	X	X
Eligibility questionnaire	X	—	—	—	—	—	—	—	—	—	—
Confirm eligibility, obtain demographics, register	X	—	—	—	—	—	—	—	—	—	—
CASI questionnaire	—	X	X	X	X	X	X	X	X	X	X
Concomitant medications	X	X	X	X	X	X	X	X	X	X	X
Social impact assessment	—	X	X	X	X	X	X	X	X	X	X
Procedure-related event assessment	—	—	—	X	X	X	X	X	X	X	X
Confirm HIV test results provided to participant	—	X	—	—	—	—	—	—	—	X	—
**Local lab assessment**											
HIV test	X	—	—	—	—	—	—	—	X	—	—
Syphilis	—	X	—	—	—	—	—	—	X	—	—
Urine NG, CT	—	X	—	—	—	—	—	—	X	—	—
HSV-2	X	—	—	—	—	—	—	—	X	—	—
CBC, differential, platelet	X	X	X	X	X	X	X	X	X	X	X
PT/PTT and INR	X	—	—	—	—	—	—	—	—	—	—
**Specimen collection[b]**											
Blood	X	X	X	X	X	X	X	X	X	X	X
Urine	—	X	—	—	—	—	—	—	X	—	—
Rectal swabs	—	X	—	—	—	—	—	—	X	—	—
Rectosigmoid biopsy[c]	—	—	—	X	—	—	—	—	—	X	—
Foreskin by circumcision[c]	—	—	—	—	—	X	—	—	—	—	—

[a] Screening may occur over the course of several contacts/visits up to and including day 0 prior to collection of all specimens required at day 0.
[b] For tests to be conducted using urine and rectal swabs, see Appendix F.
[c] Includes pre- and post-procedure visit with provider performing the procedure.

HIV VACCINE
TRIALS NETWORK

September 29, 2010

Clarification Memo #1
Protocol
Version 1.0

HVTN 914

A cohort study in Lima, Peru to evaluate the feasibility of measuring immune responses and activation levels in the foreskin and rectosigmoid mucosa in HIV-negative, uncircumcised men who have sex with men and who are at high risk for HIV acquisition

DAIDS-ES ID 11704

HIV Vaccine Trials Network (HVTN) Clinical Research Site (CRS) filing instructions

Please distribute this clarification memo to all appropriate staff members, and file with your protocol documents. Consult your local Institutional Review Board (IRB)/Ethics Committee (EC) regarding submission requirements for clarification memos.

List of changes

Item 1 Clarified in Section 9.4.2, *Real time (RT)-PCR* and Appendix F, *Laboratory procedures*: RT-PCR performed on whole blood collected in Tempus tubes 1

The changes described herein will be incorporated in the next version of Protocol HVTN 914 if it undergoes full protocol amendment at a later time.

Item 1 Clarified in Section 9.4.2, *Real time (RT)-PCR* and Appendix F, *Laboratory procedures*: RT-PCR performed on whole blood collected in Tempus tubes

Based on results of recent studies, laboratory personnel for HVTN 914 have determined that the RT-PCR assay should be performed on whole blood rather than

PBMCs. Hence, at each relevant visit, blood samples for this assay will be collected in three 3 mL Tempus tubes rather than one 10 mL ACD tube. As a consequence, the maximum blood draw at any single visit has been reduced slightly (from 83 to 82 mL) and the total blood drawn for the study as a whole has been reduced from 717 to 707 mL. These clarifications are shown below (deletion shown by strikethrough; added text in **bold underline**).

A Clarified in Section 9.4.2, *Real time (RT)-PCR*: Assay performed on whole blood rather than PBMCs

Revised:

9.4.2 Real time (RT)-PCR

RNA isolated from ~~PBMCs~~ **whole blood**, foreskin tissue, rectal biopsies, and sigmoid biopsies will be used to measure bulk levels of chemokine receptors CCR5 and CXCR4, and their ligands RANTES, MIP1α, MIP1β, and SDF-1 *in situ*. Other markers of cellular activation (eg, Ki67, TNF-α) and proteins promoting innate antiviral protection (eg, langerin, APOBEC3, and TRIM5α) may be analyzed.

B Clarified in Appendix F, *Laboratory procedures*: Tube type and blood draw total for RT-PCR assay

See attached Appendix F, *Laboratory procedures.*

Appendix F: Laboratory procedures

					Tube volume (mL)											
				Visit:	**1**	**2**	**3**	**4**	**5**	**6**	**7**	**8**	**9**	**10**	**11**	
				Day:		D0	D7	D14	D21	D28	D35	D70	D182	D189	D196	
				Week:		W0	W1	W2	W3	W4	W5	W10	W26	W27	W28	
**Description**	**Processing location**	**Intermediary processing/ storage location**	**Assay location**	**Tube**	Screening	Baseline 1	Baseline 2									**Total**
BLOOD COLLECTION																
Screening, diagnostic or safety assays																
HIV test	Local lab	Local lab	Local lab	SST	5	—	—	—	—	—	—	—	3	—	—	**8**
Syphilis	Local lab	Local lab	Local lab	SST	—	10	—	—	—	—	—	—	3	—	—	**13**
HSV-2	Local lab	Local lab	Local lab	SST	5	_	—	—	—	—	—	—	4	—	—	**9**
CBC/ Diff/ platelets	Local lab	Local lab	Local lab	EDTA	3	3	3	3	3	3	3	3	3	3	3	**33**
PT/PTT	Local lab	Local lab	Local lab	Citrate	4	—	—	—	—	—	—	—	—	—	—	**4**
Immunogenicity assays																
Flow cytometry and ICS (fresh PBMCs)	Local lab	NMRCD	FHCRC	ACD	—	—	—	20	—	20	—	—	—	20	—	**60**
Flow cytometry and ICS (frozen PBMCs)	Local lab	CSR	FHCRC	ACD	—	50	50	40	50	50	50	50	50	50	50	**490**
RT-PCR	Local lab	CSR	FHCRC	Tempus	—	9	9	9	9	9	9	9	9	9	9	**90**
Luminex multiplex assay / ELISA	Local lab	CSR	FHCRC	ACD	—	z	z	z	z	z	z	z	z	z	z	**0**
**Visit Total**					**17**	**72**	**62**	**72**	**62**	**82**	**62**	**62**	**72**	**82**	**62**	**707**
**Maximum 56-Day total**					**17**	**89**	**151**	**243**	**305**	**417**	**479**	**390**	**72**	**174**	**236**	
URINE COLLECTION																
NG/CT	Local lab	Local lab	Local lab		—	X	—	—	—	—	—	—	X	—	—	
RECTAL SWAB																
NG/CT	Local lab	FHCRC	KCPHD		—	X	—	—	—	—	—	—	X	—	—	
CIRCUMCISION																
Foreskin	NMRCD	NMRCD	FHCRC		—	—	—	—	—	X	—	—	—	—	—	
RECTOSIGMOID BIOPSY																
Rectosigmoid tissue	NMRCD	NMRCD	FHCRC		—	—	—	X	—	—	—	—	—	X	—	

CSR = Central specimen repository
HVTN Laboratory Program includes FHCRC = Fred Hutchinson Cancer Research Center (Seattle, Washington, USA)
Non-HVTN laboratories: NMRCD = Naval Medical Research Center Detachment (Lima, Peru); KCPHD = King County Public Health Department (Seattle, Washington, USA)
Screening may occur over the course of several contacts/visits up to and including day 0 prior to collection of all specimens required at day 0.
Local labs may assign appropriate alternative tube types for locally performed tests.
HSV-2 and syphilis testing at week 26 will be conducted only on participants who test negative at Visit 2.
At Visit 1 (screening), blood for HIV and HSV-2 testing will be collected in a single 10mL SST tube. At Visit 2 (Day 0), blood for syphilis testing will be drawn using a single 10mL SST tube. At Visit 9 (D182), blood for HIV, syphilis, and HSV-2 testing will be collected in a single 10 mL SST tube.
z = 5mL of plasma will be taken from ACD tubes during PBMC processing at the site processing lab.
Two baseline collections (at Visits 2 and 3) will be done to examine intra-person difference.
Maximum 56-day total for visits 6, 7, and 8 include 50mL of maximum estimated blood loss resulting from rectosigmoid biopsies and circumcision.
Maximum 56-day total for visits 4, 5, 10, and 11 includes 20mL of maximum estimated blood loss resulting from rectosigmoid biopsy.

Protocol modification history

Protocol modifications are made to HVTN protocols via clarification memos, letters of amendment, or full protocol amendments. HVTN protocols are modified and distributed according to the standard HVTN procedures as described in the HVTN *Manual of Operations* (MOP) (Organization and Policy>Vaccine Selection and Protocol Development).

The table below describes the version history of, and modifications to, Protocol HVTN 914.

Date	Protocol version	Protocol modification	Summary of modifications
September 29, 2010	Version 1.0	Clarification Memo 1	Item 1 Clarified in Section 9.4.2, *Real time (RT)-PCR* and Appendix F, *Laboratory procedures*: RT-PCR performed on whole blood collected in Tempus tubes
August 16. 2010	Version 1.0	Original protocol	NA

HIV VACCINE
TRIALS NETWORK

December 8, 2010

Clarification Memo #2

Version 1.0

HVTN 914

A cohort study in Lima, Peru to evaluate the feasibility of measuring immune responses and activation levels in the foreskin and rectosigmoid mucosa in HIV-negative, uncircumcised men who have sex with men and who are at high risk for HIV acquisition

DAIDS-ES ID 11704

HIV Vaccine Trials Network (HVTN) Clinical Research Site (CRS) filing instructions

Please distribute this clarification memo to all appropriate staff members, and file with your protocol documents. Consult your local Institutional Review Board (IRB)/Ethics Committee (EC) regarding submission requirements for clarification memos.

List of changes

Item 1 Clarified in Section 7.1, *Inclusion criteria*: INR criterion 2

Item 2 Clarified in Appendix F, *Laboratory procedures*: Processing and assay locations for NG/CT urine specimens 2

Item 3 Corrected in Appendix G, *Procedures at CRS*: Abbreviated physical exams at Weeks 0 and 2 2

Item 4 Updated in Protocol team list: Statistician, Clinical trials manager, and Project manager 2

The changes described herein will be incorporated in the next version of Protocol HVTN 914 if it undergoes full protocol amendment at a later time.

Item 1 Clarified in Section 7.1, *Inclusion criteria*: INR criterion

A portion of the International Normalized Ration (INR) inclusion criterion was omitted inadvertently in Inclusion Criterion 14 in Section 7.1 of HVTN 914 and the inclusion criterion, as written, would permit enrollment of persons at risk for bleeding disorders. The corrected INR inclusion criterion is shown below (added text in **bold underline**).

Revised:

14. **Prothrombin time (PT)** or **partial thromboplastin time (PTT)** ≤ 1.25 institutional upper limit of normal; **International Normalized Ratio (INR)** ≤ 1.5 **institutional upper limit of normal**

Item 2 Clarified in Appendix F, *Laboratory procedures*: Processing and assay locations for NG/CT urine specimens

As shown in the attached revised Appendix F, *Laboratory procedures*, the Intermediary processing/storage location and the assay location for NG/CT urine specimens have been changed from "Local lab" to "FHCRC" and "KCPHD" respectively. These locations now match those for NG/CT rectal swabs.

Item 3 Corrected in Appendix G, *Procedures at CRS*: Abbreviated physical exams at Weeks 0 and 2

Contrary to standard HVTN clinical trial practice, which is to schedule a physical exam (either full or abbreviated) at all post-enrollment clinic visits, in HVTN 914 the "Abbreviated physical exam" was inadvertently omitted at Weeks 0 and 2 in Appendix G, *Procedures at CRS*. This procedure has been restored in the attached corrected Appendix G.

Item 4 Updated in Protocol team list: Statistician, Clinical trials manager, and Project manager

The protocol team roster has been updated as shown below.

Previous:

**Statistician**	Yunda Huang, PhD<br>SCHARP, FHCRC<br>206-667-5780<br>yunda@scharp.org
**Clinical trials manager**	Niles Eaton<br>HVTN Core, FHCRC
**Project manager**	Heather Hildebrant<br>SCHARP, FHCRC

Revised:

**Statistician**	Raphael Gottardo, PhD<br>SCHARP, FHCRC<br>206-667-4076<br>rgottard@scharp.org
**Clinical trials manager**	Tamra Madenwald<br>HVTN Core, FHCRC
**Project manager**	Gina Escamilla<br>SCHARP, FHCRC

Appendix F: Laboratory procedures

					Tube volume (mL)											
				Visit:	**1**	**2**	**3**	**4**	**5**	**6**	**7**	**8**	**9**	**10**	**11**	
				Day:		D0	D7	D14	D21	D28	D35	D70	D182	D189	D196	
				Week:		W0	W1	W2	W3	W4	W5	W10	W26	W27	W28	
**Description**	**Processing location**	**Intermediary processing/ storage location**	**Assay location**	**Tube**	Screening	Baseline 1	Baseline 2									**Total**
BLOOD COLLECTION																
Screening, diagnostic or safety assays																
HIV test	Local lab	Local lab	Local lab	SST	5	—	—	—	—	—	—	—	3	—	—	**8**
Syphilis	Local lab	Local lab	Local lab	SST	—	10	—	—	—	—	—	—	3	—	—	**13**
HSV-2	Local lab	Local lab	Local lab	SST	5	_	—	—	—	—	—	—	4	—	—	**9**
CBC/ Diff/ platelets	Local lab	Local lab	Local lab	EDTA	3	3	3	3	3	3	3	3	3	3	3	**33**
PT/PTT	Local lab	Local lab	Local lab	Citrate	4	—	—	—	—	—	—	—	—	—	—	**4**
Immunogenicity assays																
Flow cytometry and ICS (fresh PBMCs)	Local lab	NMRCD	FHCRC	ACD	—	—	—	20	—	20	—	—	—	20	—	**60**
Flow cytometry and ICS (frozen PBMCs)	Local lab	CSR	FHCRC	ACD	—	50	50	40	50	50	50	50	50	50	50	**490**
RT-PCR	Local lab	CSR	FHCRC	Tempus	—	9	9	9	9	9	9	9	9	9	9	**90**
Luminex multiplex assay / ELISA	Local lab	CSR	FHCRC	ACD	—	z	z	z	z	z	z	z	z	z	z	**0**
**Visit Total**					**17**	**72**	**62**	**72**	**62**	**82**	**62**	**62**	**72**	**82**	**62**	**707**
**Maximum 56-Day total**					**17**	**89**	**151**	**243**	**305**	**417**	**479**	**390**	**72**	**174**	**236**	
URINE COLLECTION																
NG/CT	Local lab	FHCRC	KCPHD		—	X	—	—	—	—	—	—	X	—	—	
RECTAL SWAB																
NG/CT	Local lab	FHCRC	KCPHD		—	X	—	—	—	—	—	—	X	—	—	
CIRCUMCISION																
Foreskin	NMRCD	NMRCD	FHCRC		—	—	—	—	—	X	—	—	—	—	—	
RECTOSIGMOID BIOPSY																
Rectosigmoid tissue	NMRCD	NMRCD	FHCRC		—	—	—	X	—	—	—	—	—	X	—	

CSR = Central specimen repository
HVTN Laboratory Program includes FHCRC = Fred Hutchinson Cancer Research Center (Seattle, Washington, USA)
Non-HVTN laboratories: NMRCD = Naval Medical Research Center Detachment (Lima, Peru); KCPHD = King County Public Health Department (Seattle, Washington, USA)
Screening may occur over the course of several contacts/visits up to and including day 0 prior to collection of all specimens required at day 0.
Local labs may assign appropriate alternative tube types for locally performed tests.
HSV-2 and syphilis testing at week 26 will be conducted only on participants who test negative at Visit 2.
At Visit 1 (screening), blood for HIV and HSV-2 testing will be collected in a single 10mL SST tube. At Visit 2 (Day 0), blood for syphilis testing will be drawn using a single 10mL SST tube. At Visit 9 (D182), blood for HIV, syphilis, and HSV-2 testing will be collected in a single 10 mL SST tube.
z = 5mL of plasma will be taken from ACD tubes during PBMC processing at the site processing lab.
Two baseline collections (at Visits 2 and 3) will be done to examine intra-person difference.
Maximum 56-day total for visits 6, 7, and 8 include 50mL of maximum estimated blood loss resulting from rectosigmoid biopsies and circumcision.
Maximum 56-day total for visits 4, 5, 10, and 11 includes 20mL of maximum estimated blood loss resulting from rectosigmoid biopsy.

Appendix G: Procedures at CRS

Visit:	**01**[a]	**02**	**03**	**04**	**05**	**06**	**07**	**08**	**09**	**10**	**11**
Day:		D0	D7	D14	D21	D28	D35	D70	D182	D189	D196
Week:		W0	W1	W2	W3	W4	W5	W10	W26	W27	W28
Procedure	Scr.										
**Study procedures**											
Signed informed consent	X	—	—	—	—	—	—	—	—	—	—
Assessment of understanding	X	—	—	—	—	—	—	—	—	—	—
Medical history	X	—	—	—	—	—	—	—	—	—	—
Complete physical exam	X	—	—	—	—	—	—	—	—	—	—
Abbreviated physical exam	—	X	X	X	X	X	X	X	X	X	X
Assessment of STI signs and symptoms	X	—	X	—	X	—	X	X	X	—	X
Evaluation to confirm proper healing	—	—	—	—	X	—	—	X	—	—	X
Pre- and post-HIV test counseling	X	—	—	—	—	—	—	—	X	—	—
Risk reduction counseling	X	X	X	X	X	X	X	X	X	X	X
Counseling on procedure-specific safety criteria	X	X	X	X	X	X	X	X	X	X	X
Eligibility questionnaire	X	—	—	—	—	—	—	—	—	—	—
Confirm eligibility, obtain demographics, register	X	—	—	—	—	—	—	—	—	—	—
CASI questionnaire	—	X	X	X	X	X	X	X	X	X	X
Concomitant medications	X	X	X	X	X	X	X	X	X	X	X
Social impact assessment	—	X	X	X	X	X	X	X	X	X	X
Procedure-related event assessment	—	—	—	X	X	X	X	X	X	X	X
Confirm HIV test results provided to participant	—	X	—	—	—	—	—	—	—	X	—
**Local lab assessment**											
HIV test	X	—	—	—	—	—	—	—	X	—	—
Syphilis	—	X	—	—	—	—	—	—	X	—	—
Urine NG, CT	—	X	—	—	—	—	—	—	X	—	—
HSV-2	X	—	—	—	—	—	—	—	X	—	—
CBC, differential, platelet	X	X	X	X	X	X	X	X	X	X	X
PT/PTT and INR	X	—	—	—	—	—	—	—	—	—	—
**Specimen collection**[b]											
Blood	X	X	X	X	X	X	X	X	X	X	X
Urine	—	X	—	—	—	—	—	—	X	—	—
Rectal swabs	—	X	—	—	—	—	—	—	X	—	—
Rectosigmoid biopsy[c]	—	—	—	X	—	—	—	—	—	X	—
Foreskin by circumcision[c]	—	—	—	—	—	X	—	—	—	—	—

[a] Screening may occur over the course of several contacts/visits up to and including day 0 prior to collection of all specimens required at day 0.
[b] For tests to be conducted using urine and rectal swabs, see Appendix F.
[c] Includes pre- and post-procedure visit with provider performing the procedure.

Protocol modification history

Protocol modifications are made to HVTN protocols via clarification memos, letters of amendment, or full protocol amendments. HVTN protocols are modified and distributed according to the standard HVTN procedures as described in the HVTN *Manual of Operations* (MOP) (Organization and Policy>Vaccine Selection and Protocol Development).

The table below describes the version history of, and modifications to, Protocol HVTN 914.

Date	Protocol version	Protocol modification	Summary of modifications
December 8, 2010	Version 1.0	Clarification Memo 2	Item 1 Clarified in Section 7.1, *Inclusion criteria*: INR criterion<br>Item 2 Clarified in Appendix F, *Laboratory procedures*: Processing and assay locations for NG/CT urine specimens<br>Item 3 Corrected in Appendix G, *Procedures at CRS*: Abbreviated physical exams at Weeks 0 and 2<br>Item 4 Updated in Protocol team list: Statistician, Clinical trials manager, and Project manager
September 29, 2010	Version 1.0	Clarification Memo 1	Item 1 Clarified in Section 9.4.2, *Real time (RT)-PCR* and Appendix F, *Laboratory procedures*: RT-PCR performed on whole blood collected in Tempus tubes
August 16. 2010	Version 1.0	Original protocol	NA

November 29, 2011

Letter of Amendment #1

Version 1.0

HVTN 914

A cohort study in Lima, Peru to evaluate the feasibility of measuring immune responses and activation levels in the foreskin and rectosigmoid mucosa in HIV-negative, uncircumcised men who have sex with men and who are at high risk for HIV acquisition

DAIDS-ES ID 11704

HIV Vaccine Trials Network (HVTN) Clinical Research Site (CRS) filing instructions

The following information impacts the HVTN 914 study and must be forwarded to your Institutional Review Board (IRB)/Ethics Committee (EC) and any other applicable Regulatory Entity (RE) as soon as possible for their information and review. Their approval is required before implementation.

Upon receiving final IRB/EC and any other applicable RE approvals, sites are required to submit a Letter of Amendment (LOA) registration packet to the DAIDS Protocol Registration Office (PRO) at the Regulatory Support Center (RSC). Sites will receive a Registration Notification for the LOA once the DAIDS PRO verifies that all the required LOA registration documents have been received and are complete. A Registration Notification from the DAIDS PRO is not required prior to implementing the LOA. A copy of the Registration Notification along with this letter of amendment and any IRB/EC correspondence should be retained in the site's regulatory files.

For additional information on the registration process and specific documents required for LOA registration, refer to the current version of the DAIDS Protocol Registration Manual.

The following information affects the sample informed consent. Your IRB/EC will be responsible for determining the process of informing study participants of the contents of this letter of amendment.

List of changes

Item 1 Clarified in Sections 3, 6.1, and 7.3: Sample size, participant withdrawal, replacement, and termination 2

Item 2 Clarified in Appendix A, *Sample informed consent form*: Enemas prior to flexible sigmoidoscopies may occur at home or in the clinic 3

Item 3 Clarified in Appendix A, *Sample informed consent form*: Samples shipped out of country for analysis 3

Item 4 Clarified in Appendix F, *Laboratory procedures*: Resolution of indeterminate HSV-2 test results 4

The changes described herein will be incorporated in the next version of Protocol HVTN 914 if it undergoes full protocol amendment at a later time.

Item 1 Clarified in Sections 3, 6.1, and 7.3: Sample size, participant withdrawal, replacement, and termination

Section 6.1 of HVTN 914 indicates that participants who withdraw from the study before completing week 4 procedures (esp. circumcision) will be replaced. The section also indicates that data from all enrolled participants, including those terminated early, will be included in the evaluation of study objectives, as appropriate. The protocol, however, does not provide specific direction regarding the disposition of such participants. In order to clarify this matter, Sections 3, 6.1, and 7.3 have been revised as shown below (deletions shown by ~~strikethrough~~; added text in **bold underline**).

A Revised in Section 3, *Overview*

Revised:

Participants

30 healthy, HIV-seronegative, uncircumcised men, aged 21 to 30 years, who have sex with men (MSM) and who are at high risk for acquisition of HIV **(see Section 6.1)**

B Revised in Section 6.1, *Accrual and sample size calculations*

Revised:

This cohort study will enroll 30 HIV-seronegative, uncircumcised 21-30 year old MSM at high risk of HIV infection in Lima, Peru. ~~These subjects~~ **Enrollees** will be equally stratified into 6 groups defined by primary sexual preferences (insertive, versatile, or receptive) and baseline HSV-2 status (positive or negative). Participants ~~withdrawing from the study prior to completion of all study procedures~~ **who do not complete the rectosigmoid biopsy planned for week 2 and the circumcision** planned for week 4 **are not considered fully evaluable and** will be replaced with new eligible volunteers belonging to the same stratification group **(see Section 7.3)**. ~~However, a~~**A**ll data from enrolled participants, including those **who withdraw or who are** terminated from the study early, will be included in the evaluation of all study objectives, when appropriate.

C Added in Section 7.3, *Participant termination from the study*

The following text has been added to the end of Section 7.3.

Added:

In addition, failure to complete certain critical study procedures will result in participant termination. For instance,

- **Participants who do not complete the rectosigmoid biopsy scheduled for week 2 will be terminated from the study. Such participants will be replaced with new eligible volunteers belonging to the same stratification group.**
- **Participants who complete the rectosigmoid biopsy planned for week 2 but who cannot or decline to complete the circumcision scheduled for week 4 should complete the safety visit scheduled for week 3, following which they should be terminated from the study. Such participants will be replaced with new eligible volunteers belonging to the same stratification group.**

Participants who complete the circumcision scheduled for week 4 should, if possible, complete the entire schedule of study visits and procedures. If such participants subsequently withdraw or are lost to follow-up, they will not be replaced.

Item 2 Clarified in Appendix A, *Sample informed consent form*: Enemas prior to flexible sigmoidoscopies may occur at home or in the clinic

Section 9 of Appendix A indicates that study participants will be given two enemas to cleanse the colon prior to flexible sigmoidoscopy. The phrasing inadvertently omitted the possibility that one or both of these enemas may be administered in the clinic prior to the flexible sigmoidoscopy procedure. For clarity, the phrase “at home” has been removed, as shown below (deletion shown by ~~strikethrough~~).

Revised:

The colon and rectum must be completely empty for flexible sigmoidoscopy, so you will be asked to drink only clear liquids for 12 to 24 hours beforehand. You will also be given an enema solution, which is a liquid that washes out the intestines, to apply twice before the procedure~~ at home~~.

Item 3 Clarified in Appendix A, *Sample informed consent form*: Samples shipped out of country for analysis

Section 11 of the Appendix A, *Sample informed consent form,* indicates that extra samples donated by study participants may be shared with researchers in other countries. To clarify that samples collected within this study also may be shipped out of country for analysis (eg as indicated in Item 3 below), a sentence has been added to the penultimate paragraph in Section 5 of Appendix A, as shown below (added text in **bold underline**).

Revised:

More detailed information about rectal swabs, rectal and colon tissue collection, and circumcision follows. **Some samples collected through these procedures may be sent to other countries for analysis.**

Item 4 Clarified in Appendix F, *Laboratory procedures*: Resolution of indeterminate HSV-2 test results

The HVTN Laboratory Program has determined that, in order to resolve indeterminate "Local lab" HSV-2 test results, samples resulting in indeterminate results should be forwarded to the University of Washington Virology Laboratory (Seattle, Washington, USA) for Western blot testing. A footnote to this effect has been added below Appendix F, *Laboratory procedures*, as shown below and in the attached Appendix F (added text in **bold underline)**.

Added:

Samples from volunteers with indeterminate HSV-2 test results to be forwarded to the University of Washington Virology Laboratory (Seattle, Washington, USA) for Western blot testing.

Appendix F: Laboratory procedures

					Tube volume (mL)											
				Visit:	**1**	**2**	**3**	**4**	**5**	**6**	**7**	**8**	**9**	**10**	**11**	
				Day:		D0	D7	D14	D21	D28	D35	D70	D182	D189	D196	
				Week:		W0	W1	W2	W3	W4	W5	W10	W26	W27	W28	
**Description**	**Processing location**	**Intermediary processing/ storage location**	**Assay location**	**Tube**	Screening	Baseline 1	Baseline 2									**Total**
BLOOD COLLECTION																
Screening, diagnostic or safety assays																
HIV test	Local lab	Local lab	Local lab	SST	5	—	—	—	—	—	—	—	3	—	—	**8**
Syphilis	Local lab	Local lab	Local lab	SST	—	10	—	—	—	—	—	—	3	—	—	**13**
HSV-2	Local lab	Local lab	Local lab	SST	5	_	—	—	—	—	—	—	4	—	—	**9**
CBC/ Diff/ platelets	Local lab	Local lab	Local lab	EDTA	3	3	3	3	3	3	3	3	3	3	3	**33**
PT/PTT	Local lab	Local lab	Local lab	Citrate	4	—	—	—	—	—	—	—	—	—	—	**4**
Immunogenicity assays																
Flow cytometry and ICS (fresh PBMCs)	Local lab	NMRCD	FHCRC	ACD	—	—	—	20	—	20	—	—	—	20	—	**60**
Flow cytometry and ICS (frozen PBMCs)	Local lab	CSR	FHCRC	ACD	—	50	50	40	50	50	50	50	50	50	50	**490**
RT-PCR	Local lab	CSR	FHCRC	Tempus	—	9	9	9	9	9	9	9	9	9	9	**90**
Luminex multiplex assay / ELISA	Local lab	CSR	FHCRC	ACD	—	z	z	z	z	z	z	z	z	z	z	**0**
**Visit Total**					**17**	**72**	**62**	**72**	**62**	**82**	**62**	**62**	**72**	**82**	**62**	**707**
**Maximum 56-Day total**					**17**	**89**	**151**	**243**	**305**	**417**	**479**	**390**	**72**	**174**	**236**	
URINE COLLECTION																
NG/CT	Local lab	FHCRC	KCPHD		—	X	—	—	—	—	—	—	X	—	—	
RECTAL SWAB																
NG/CT	Local lab	FHCRC	KCPHD		—	X	—	—	—	—	—	—	X	—	—	
CIRCUMCISION																
Foreskin	NMRCD	NMRCD	FHCRC		—	—	—	—	—	X	—	—	—	—	—	
RECTOSIGMOID BIOPSY																
Rectosigmoid tissue	NMRCD	NMRCD	FHCRC		—	—	—	X	—	—	—	—	—	X	—	

CSR= Central specimen repository

HVTN Laboratory Program includes FHCRC = Fred Hutchinson Cancer Research Center (Seattle, Washington, USA)

Non-HVTN laboratories: NMRCD = Naval Medical Research Center Detachment (Lima, Peru); KCPHD = King County Public Health Department (Seattle, Washington, USA)

Screening may occur over the course of several contacts/visits up to and including day 0 prior to the collection of all specimens required at Day 0

Local labs may assign appropriate alternative tube types for locally performed tests.

Samples from volunteers with indeterminate HSV-2 test results to be forwarded to the University of Washington Virology Laboratory (Seattle, Washington, USA) for Western blot testing.

HSV-2 and syphilis testing at week 25 will only be conducted on participants who respectively test negative at Visit 2

At Visit 1 (screening), blood for HIV and HSV-2 testing will be collected in a single 10mL SST tube. At Visit 2 (Day 0), blood for syphilis testing will be drawn using a single 10mL SST tube. At Visit 9 (D182), blood for HIV, syphilis, and HSV-2 testing will be collected in a single 10 mL SST tube.

z = 5mL of plasma will be taken from ACD tubes during PBMC processing at the site-affiliated lab

Two baseline collections at Visits 2 and 3 will be done to examine intra-person difference.

Maximum 56-day totals for Visits 6, 7, and 8 include 50mL of maximum estimated blood loss resulting from rectosigmoid biopsies and circumcision.

Maximum 56-day total for Visits 4, 5, 10, and 11 include 20mL of maximum estimated blood loss resulting from rectosigmoid biopsy.

Protocol modification history

Protocol modifications are made to HVTN protocols via clarification memos, letters of amendment, or full protocol amendments. HVTN protocols are modified and distributed according to the standard HVTN procedures as described in the HVTN Manual of Operations (MOP) (Organization and Policy>Vaccine Selection and Protocol Development).

The table below describes the version history of, and modifications to, Protocol HVTN 914.

Date	Protocol version	Protocol modification	Summary of modifications
November 29, 2011	Version 1.0	Letter of Amendment 1	Item 1 Clarified in Sections 3, 6.1, and 7.3: Sample size, participant withdrawal, replacement, and termination<br>Item 2 Clarified in Appendix A, *Sample informed consent form*: Enemas prior to flexible sigmoidoscopies may occur at home or in the clinic<br>Item 3 Clarified in Appendix A, *Sample informed consent form*: Samples shipped out of country for analysis<br>Item 4 Clarified in Appendix F, *Laboratory procedures*: Resolution of indeterminate HSV-2 test results
December 8, 2010	Version 1.0	Clarification Memo 2	Item 1 Clarified in Section 7.1, *Inclusion criteria*: INR criterion<br>Item 2 Clarified in Appendix F, *Laboratory procedures*: Processing and assay locations for NG/CT urine specimens<br>Item 3 Corrected in Appendix G, *Procedures at CRS*: Abbreviated physical exams at Weeks 0 and 2<br>Item 4 Updated in Protocol team list: Statistician, Clinical trials manager, and Project manager
September 29, 2010	Version 1.0	Clarification Memo 1	Item 1 Clarified in Section 9.4.2, *Real time (RT)-PCR* and Appendix F, *Laboratory procedures*: RT-PCR performed on whole blood collected in Tempus tubes
August 16. 2010	Version 1.0	Original protocol	NA

HIV VACCINE
TRIALS NETWORK

January 4, 2012

Letter of Amendment #2

Version 1.0

HVTN 914

A cohort study in Lima, Peru to evaluate the feasibility of measuring immune responses and activation levels in the foreskin and rectosigmoid mucosa in HIV-negative, uncircumcised men who have sex with men and who are at high risk for HIV acquisition

DAIDS-ES ID 11704

HIV Vaccine Trials Network (HVTN) Clinical Research Site (CRS) filing instructions

The following information impacts the HVTN 914 study and must be forwarded to your Institutional Review Board (IRB)/Ethics Committee (EC) and any other applicable Regulatory Entity (RE) as soon as possible for their information and review. Their approval is required before implementation.

Upon receiving final IRB/EC and any other applicable RE approvals, sites are required to submit a Letter of Amendment (LOA) registration packet to the DAIDS Protocol Registration Office (PRO) at the Regulatory Support Center (RSC). Sites will receive a Registration Notification for the LOA once the DAIDS PRO verifies that all the required LOA registration documents have been received and are complete. A Registration Notification from the DAIDS PRO is not required prior to implementing the LOA. A copy of the Registration Notification along with this letter of amendment and any IRB/EC correspondence should be retained in the site's regulatory files.

For additional information on the registration process and specific documents required for LOA registration, refer to the current version of the DAIDS Protocol Registration Manual.

The following information affects the sample informed consent. Your IRB/EC will be responsible for determining the process of informing study participants of the contents of this letter of amendment.

List of changes

Item 1 Revised in Sections 5.3, 8.6, 8.7, 9.4.5, and Appendices A, B, F, and G: HIV surveillance enhanced 2

The changes described herein will be incorporated in the next version of Protocol HVTN 914 if it undergoes full protocol amendment at a later time.

Item 1 Revised in Sections 5.3, 8.6, 8.7, 9.4.5, and Appendices A, B, F, and G: HIV surveillance enhanced

Protocol HVTN 914 states in Section 7.3 that HIV-infected participants will be terminated from the study. HIV testing was specified in the protocol at screening and at week 26. This has proven insufficient. In order to monitor for HIV infection among study participants more effectively, HIV tests have been added prior to invasive study procedures at Visit 4 (Week 2, rectosigmoid biopsy) and Visit 6 (Week 4, circumcision); similarly, the HIV test at Visit 9 has been moved to Visit 10, immediately prior to the second rectosigmoid biopsy. To further enhance surveillance for HIV infection, an HIV test has been added at Visit 8 (Week 10) and an additional visit with an HIV test has been added at Week 16; this new visit has been designated Visit 50. Appendix A, *Sample informed consent form* and Appendix B, *Table of procedures (for sample informed consent form)*, have been revised to reflect this additional visit. In addition, in order to determine more precisely the time of infection for study participants who become HIV-infected, provision has been added in Section 5.3 (*Exploratory objective and endpoints*), in new Section 9.4.5, and in footnotes to Appendix F (*Laboratory* procedures) for testing stored plasma samples using HIV RNA PCR for participants who become HIV-infected. Additional minor corrections and clarifications in Appendix F are detailed in Items 1H and 1I. These revisions are shown below (deletions shown by ~~strikethrough~~, added text in **<u>bold underline</u>**).

A HIV RNA PCR testing added to exploratory endpoint 1 in Section 5.3

Revised:

Exploratory objective 1:

To assess the HIV risk associated with sigmoidoscopy and circumcision procedures in sexually active MSM.

Exploratory endpoints 1:

Pre- and post-procedure (1) HIV risk behaviors and (2) levels of activation markers in PBMCs that are associated with vulnerability to HIV infection.

<u>Time of infection for HIV-infected study participants as determined by HIV RNA PCR.</u>

B Negative HIV test required prior to procedure in Section 8.6, *Flexible sigmoidoscopy*

Revised:

8.6 Flexible sigmoidoscopy

Flexible sigmoidoscopy will be conducted at week 2 and week 27 to obtain rectosigmoid mucosal specimens for evaluation of mucosal anatomy, HIV target cells, innate resistance factors, and antigen-specific responses. Per individual, up to 25 sigmoid biopsy samples will be taken and up to 4 rectal biopsy samples will be taken. Fewer samples may be taken based on the judgment of the performing clinician. **Negative HIV test results must be obtained at visit prior to initiating this procedure.**

C Negative HIV test required prior to procedure in Section 8.7, *Circumcision*

Revised:

8.7 Circumcision

Circumcision will be conducted during week 4 to obtain foreskin samples for evaluation of mucosal anatomy, HIV target cells, innate resistance factors, and antigen specific responses. **Negative HIV test results must be obtained at visit prior to initiating this procedure.**

D Retrospective HIV RNA PCR testing added as new Section 9.4.5

Added:

9.4.5 HIV RNA PCR testing

For participants who become HIV-infected, the earliest date of detectable HIV RNA will be assessed via HIV RNA PCR testing of stored plasma samples.

E Number of visits revised in Appendix A, *Sample informed consent form*

Revised:

4. You will come to the clinic about ~~10~~ 11 times over a period of about 7 months.

Site: Insert range of visit lengths.

Visits can last from [#] to [#] hours.

You may have to come for more visits if you have a laboratory or health issue.

F Visit and study procedures added in Appendix B, *Table of procedures (for sample informed consent form)*

Revised:

Appendix B: Table of procedures (for sample informed consent form)

	Screening	Weeks after enrollment visit										
		W 0	W 1	W 2	W 3	W 4	W 5	W 10	**W 16**	W 26	W 27	W 28
Medical history	√											
Complete physical	√											
Brief physical		√	√	√	√	√	√	√	√	√	√	√
HIV testing/counseling	√			√		√		√	√	~~√~~	√	
Interview/questionnaire	√	√			√	√	√	√	√	√	√	√
Computer questionnaire		√	√	√	√	√	√	√	√	√	√	√
Risk reduction counseling	√	√	√	√	√	√	√	√	√	√	√	√
Blood drawn	√	√	√	√	√	√	√	√	√	√	√	√
Urine test		√								√		
Rectal swabs		√								√		
Rectal/colon biopsy				√							√	
Circumcision						√						

G Additional visit and study procedures added in Appendix F, *Laboratory procedures*

HIV tests have been added at Visits 4, 6, 8, and new Visit 50. The Visit 9 HIV test has been moved to visit 10. In addition, the assay location for HIV PCR testing has been added to the footnote listing HVTN Laboratory Program labs, a blood draw for plasma for PCR testing has been added to new Visit 50, and a footnote has been added indicating that, if necessary, stored plasma samples designated for "Luminex multiplex assay/ELISA" may be used for PCR testing to determine the timing of HIV infection. See the revised table and footnotes below (added footnote text in **bold underline**).

H Blood draw volumes and tube sizes updated in Appendix F, *Laboratory procedures*

Some time ago, the HVTN Laboratory Program changed its convention for blood volumes from listing nominal tube sizes to listing the actual amount of blood that can be drawn into each type of tube. Blood volumes in Appendix F have been revised per to the new convention. In addition, a column for tube sizes has been added, blood draw volumes for some procedures have been revised for consistency with the assigned tubes, and a footnote related to drawing multiple samples into a single tube has been deleted. See the revised Appendix F table below.

I Storage locations clarified in Appendix F, *Laboratory procedures*

The Fred Hutchinson Cancer Research Center (FHCRC) serves as the repository for all samples collected in HVTN 914. For consistency within the table and to avoid the appearance that CSR and FHCRC are two different locations, "CSR" has been replaced throughout the table with "FHCRC" and the footnote defining "CSR" has been deleted. See the revised Appendix F below (deletion shown by ~~strikethrough~~).

J Typographical error corrected in footnote to Appendix F, Laboratory procedures

An erroneous footnote reference to a visit at week 25 has been corrected to week 26. See the revised Appendix F below (deletion shown by ~~strikethrough~~; added text in **<u>bold underline</u>**).

K Additional visit and study procedures added in Appendix G, *Procedures at CRS*

HIV tests (and pre- and post-test counseling) have been added at Visits 4, 6, 8, and new Visit 50. Once enrolled, confirmation that test results have been provided to study participants will occur the same day the tests are performed. The rapid HIV test results are required on the day of the test and therefore the results must be conveyed to the participant the same day with confirmation occurring concurrently. The Visit 9 HIV test has been moved to Visit 10. Other procedures have been added for new Visit 50 (Week 16). See the revised table below (deletions shown by ~~strikethrough~~; added text in **<u>bold underline</u>**).

Appendix F: Laboratory procedures

						Tube volume (mL)												
					Visit:	**1**	**2**	**3**	**4**	**5**	**6**	**7**	**8**	**50**	**9**	**10**	11	
					Day:		D0	D7	D14	D21	D28	D35	D70	D112	D182	D189	D196	
					Week:		W0	W1	W2	W3	W4	W5	W10	W16	W26	W27	W28	
**Description**	**Processing location**	**Intermediary processing/ storage location**	**Assay location**	**Tube**	**Tube size (vol capacity)**	Screening	Baseline 1	Baseline 2										**Total**
BLOOD COLLECTION																		
Screening, diagnostic or safety assays																		
HIV test	Local lab	Local lab	Local lab	SST	5 mL	5	—	—	5	—	5	—	5	5	—	5	—	**30**
Syphilis	Local lab	Local lab	Local lab	SST	5 mL	—	5	—	—	—	—	—	—	—	5	—	—	**10**
HSV-2	Local lab	Local lab	Local lab	SST	5 mL	5	_	—	—	—	—	—	—	—	5	—	—	**10**
CBC/ Diff/ platelets	Local lab	Local lab	Local lab	EDTA	3 mL	3	3	3	3	3	3	3	3	—	3	3	3	**33**
PT/PTT	Local lab	Local lab	Local lab	Citrate	4 mL	4	—	—	—	—	—	—	—	—	—	—	—	**4**
Immunogenicity assays																		
Flow cytometry and ICS (fresh PBMCs)	Local lab	NMRCD	FHCRC	ACD	10 mL	—	—	—	17	—	17	—	—	—	—	17	—	**51**
Flow cytometry and ICS (frozen PBMCs)	Local lab	FHCRC	FHCRC	ACD	10 mL	—	42.5	42.5	34	42.5	42.5	42.5	42.5	—	42.5	42.5	42.5	**416.5**
RT-PCR	Local lab	FHCRC	FHCRC	Tempus	3 mL	—	9	9	9	9	9	9	9	—	9	9	9	**90**
Luminex multiplex assay / ELISA*	Local lab	FHCRC	FHCRC	ACD	10 mL	—	z	z	z	z	z	z	z	—	z	z	z	**0**
Storage																		
Plasma	Local lab	FHCRC	UW-VSL	ACD	10 mL	—	—	—	—	—	—	—	—	8.5	—	—	—	**8.5**
**Visit Total**						**17**	**59.5**	**54.5**	**68**	**54.5**	**76.5**	**54.5**	**59.5**	**13.5**	**64.5**	**76.5**	**54.5**	**653**
**Maximum 56-Day total**						**17**	**76.5**	**131**	**219**	**273.5**	**400**	**454.5**	**383**	**73**	**64.5**	**161**	**215.5**	
URINE COLLECTION																		
NG/CT	Local lab	FHCRC	KCPHD			—	X	—	—	—	—	—	—	—	X	—	—	
RECTAL SWAB																		
NG/CT	Local lab	FHCRC	KCPHD			—	X	—	—	—	—	—	—	—	X	—	—	
CIRCUMCISION																		
Foreskin	NMRCD	NMRCD	FHCRC			—	—	—	—	—	X	—	—	—	—	—	—	
RECTOSIGMOID BIOPSY																		
Rectosigmoid tissue	NMRCD	NMRCD	FHCRC			—	—	—	X	—	—	—	—	—	—	X	—	

~~CSR = Central specimen repository~~

HVTN Laboratory Program includes**:** FHCRC = Fred Hutchinson Cancer Research Center (Seattle, Washington, USA), **UW-VSL = University of Washington Virology Specialty Laboratory**

Non-HVTN laboratories: NMRCD = Naval Medical Research Center Detachment (Lima, Peru); KCPHD = King County Public Health Department (Seattle, Washington, USA)

Screening may occur over the course of several contacts/visits up to and including day 0 prior to the collection of all specimens required at Day 0

Local labs may assign appropriate alternative tube types for locally performed tests.

Samples from volunteers with indeterminate HSV-2 test results to be forwarded to the University of Washington Virology Laboratory (Seattle, Washington, USA) for Western blot testing.

HSV-2 and syphilis testing at week ~~25~~**26** will only be conducted on participants who respectively test negative at Visit 2

~~At Visit 1 (screening), blood for HIV and HSV-2 testing will be collected in a single 10mL SST tube. At Visit 2 (Day 0), blood for syphilis testing will be drawn using a single 10mL SST tube. At Visit 9 (D182), blood for HIV, syphilis, and HSV-2 testing will be collected in a single 10 mL SST tube.~~

z = 5mL of plasma will be taken from ACD tubes during PBMC processing at the site-affiliated lab

Two baseline collections at Visits 2 and 3 will be done to examine intra-person difference.

Maximum 56-day totals for Visits 6, 7, and 8 include 50mL of maximum estimated blood loss resulting from rectosigmoid biopsies and circumcision.

Maximum 56-day total for Visits 4, 5, 10, and 11 include 20mL of maximum estimated blood loss resulting from rectosigmoid biopsy.

*** If necessary, stored plasma samples may be used for PCR testing to determine the onset of HIV infection**

Appendix G: Procedures at CRS

Visit:	**01**[1]	**02**	**03**	**04**	**05**	**06**	**07**	**08**	**50**	**09**	**10**	**11**
Day:		D0	D7	D14	D21	D28	D35	D70	**D112**	D182	D189	D196
Week:		W0	W1	W2	W3	W4	W5	W10	**W16**	W26	W27	W28
Procedure	Scr.											
**Study procedures**												
Signed informed consent	X	—	—	—	—	—	—	—	—	—	—	—
Assessment of understanding	X	—	—	—	—	—	—	—	—	—	—	—
Medical history	X	—	—	—	—	—	—	—	—	—	—	—
Complete physical exam	X	—	—	—	—	—	—	—	—	—	—	—
Abbreviated physical exam	—	X	X	X	X	X	X	X	**X**	X	X	X
Assessment of STI signs and symptoms	X	—	X	—	X	—	X	X	**X**	X	—	X
Evaluation to confirm proper healing	—	—	—	—	X	—	—	X	—	—	—	X
Pre- and post-HIV test counseling	X	—	—	**X**	—	**X**	—	**X**	**X**	—	**X**	—
Risk reduction counseling	X	X	X	X	X	X	X	X	**X**	X	X	X
Counseling on procedure-specific safety criteria	X	X	X	X	X	X	X	X	**X**	X	X	X
Eligibility questionnaire	X	—	—	—	—	—	—	—	—	—	—	—
Confirm eligibility, obtain demographics, register	X	—	—	—	—	—	—	—	—	—	—	—
CASI questionnaire	—	X	X	X	X	X	X	X	**X**	X	X	X
Concomitant medications	X	X	X	X	X	X	X	X	**X**	X	X	X
Social impact assessment	—	X	X	X	X	X	X	X	**X**	X	X	X
Procedure-related event assessment	—	—	—	X	X	X	X	X	**X**	X	X	X
Confirm HIV test results provided to participant	—	X	—	**X**	—	**X**	—	**X**	**X**	—	X	—
**Local lab assessment**												
HIV test	X	—	—	**X**	—	**X**	—	**X**	**X**	~~X~~	**X**	—
Syphilis	—	X	—	—	—	—	—	—	—	X	—	—
Urine NG, CT	—	X	—	—	—	—	—	—	—	X	—	—
HSV-2	X	—	—	—	—	—	—	—	—	X	—	—
CBC, differential, platelet	X	X	X	X	X	X	X	X	—	X	X	X
PT/PTT and INR	X	—	—	—	—	—	—	—	—	—	—	—
**Specimen collection**[2]												
Blood	X	X	X	X	X	X	X	X	**X**	X	X	X
Urine	—	X	—	—	—	—	—	—	—	X	—	—
Rectal swabs	—	X	—	—	—	—	—	—	—	X	—	—
Rectosigmoid biopsy[3]	—	—	—	X	—	—	—	—	—	—	X	—
Foreskin by circumcision[c]	—	—	—	—	—	X	—	—	—	—	—	—

[1] Screening may occur over the course of several contacts/visits up to and including day 0 prior to collection of all specimens required at day 0.
[2] For tests to be conducted using urine and rectal swabs, see Appendix F.
[3] Includes pre- and post-procedure visit with provider performing the procedure.

Protocol modification history

Protocol modifications are made to HVTN protocols via clarification memos, letters of amendment, or full protocol amendments. HVTN protocols are modified and distributed according to the standard HVTN procedures as described in the HVTN Manual of Operations (MOP) (Organization and Policy>Vaccine Selection and Protocol Development).

The version history of, and modifications to, Protocol HVTN 914 are described below.

Date: January 4, 2012

Protocol version: 1.0
Protocol modification: Letter of Amendment 2

Item 1 Revised in Sections 5.3, 8.6, 8.7, 9.4.5, and Appendices A, B, F, and G: HIV surveillance enhanced

November 29, 2011

Protocol version: 1.0
Protocol modification: Letter of Amendment 1

Item 1 Clarified in Sections 3, 6.1, and 7.3: Sample size, participant withdrawal, replacement, and termination

Item 2 Clarified in Appendix A, *Sample informed consent form*: Enemas prior to flexible sigmoidoscopies may occur at home or in the clinic

Item 3 Clarified in Appendix A, *Sample informed consent form*: Samples shipped out of country for analysis

Item 4 Clarified in Appendix F, Laboratory procedures*:* Resolution of indeterminate HSV-2 test results

Date: December 8, 2010

Protocol version: 1.0
Protocol modification: Clarification Memo 2

Item 1 Clarified in Section 7.1, *Inclusion criteria*: INR criterion

Item 2 Clarified in Appendix F, *Laboratory procedures*: Processing and assay locations for NG/CT urine specimens

Item 3 Corrected in Appendix G, *Procedures at CRS*: Abbreviated physical exams at Weeks 0 and 2

Item 4 Updated in Protocol team list: Statistician, Clinical trials manager, and Project manager

Date: September 29, 2010

Protocol version: 1.0
Protocol modification: Clarification Memo 1

Item 1 Clarified in Section 9.4.2, *Real time (RT)-PCR* and Appendix F, *Laboratory procedures*: RT-PCR performed on whole blood collected in Tempus tubes

Date: August 16. 2010

Protocol version: 1.0
Protocol modification: NA

Original protocol

February 8, 2012

Letter of Amendment #3

Version 1.0

HVTN 914

A cohort study in Lima, Peru to evaluate the feasibility of measuring immune responses and activation levels in the foreskin and rectosigmoid mucosa in HIV-negative, uncircumcised men who have sex with men and who are at high risk for HIV acquisition

DAIDS-ES ID 11704

HIV Vaccine Trials Network (HVTN) Clinical Research Site (CRS) filing instructions

The following information impacts the HVTN 914 study and must be forwarded to your Institutional Review Board (IRB)/Ethics Committee (EC) and any other applicable Regulatory Entity (RE) as soon as possible for their information and review. Their approval is required before implementation. Upon receiving final IRB/EC and any other applicable Regulatory Entity (RE) approval(s) for this LoA, sites should implement the LoA immediately.

Upon receiving final IRB/EC and any other applicable RE approvals, sites are required to submit a Letter of Amendment (LOA) registration packet to the DAIDS Protocol Registration Office (PRO) at the Regulatory Support Center (RSC). Sites will receive a Registration Notification for the LOA once the DAIDS PRO verifies that all the required LOA registration documents have been received and are complete. A Registration Notification from the DAIDS PRO is not required prior to implementing the LOA. A copy of the Registration Notification along with this letter of amendment and any IRB/EC correspondence should be retained in the site's regulatory files.

For additional information on the registration process and specific documents required for LOA registration, refer to the current version of the DAIDS Protocol Registration Manual.

The following information may also affect the sample informed consent. Your IRB/EC will be responsible for determining the process of informing study participants of the contents of this letter of amendment.

List of changes

Item 1 Clarified in Section 5.2, *Secondary objectives and endpoints* Section 9.4, *Immunological assays*: Humoral immune activity in foreskin.. 2

The changes described herein will be incorporated in the next version of Protocol HVTN 914 if it undergoes full protocol amendment at a later time.

Item 1 Clarified in Section 5.2, *Secondary objectives and endpoints* Section 9.4, *Immunological assays*: Humoral immune activity in foreskin

In the secondary objective 5.2, HVTN914 aims to characterize mucosal immune responses in PBMCs, foreskin, and rectosigmoid mucosa. In order to more fully understand immune system activity in the foreskin, which may help account for both vulnerability and resistance to infection, studies of B cells and immunoglobulins have been added to Secondary endpoints 1 in Section 5.2 and to the immunological assays described in Section 9.4. These changes are shown below (added text in **bold underline**).

These studies use samples already being collected in the study. This modification has no effect on participant safety or experience of the study.

A Revised in Section 5.2

Revised:

Secondary endpoints 1:

Density, activation, and location of HIV target cells **and B cells** in foreskin and rectosigmoid mucosa by immunofluorescence microscopy and flow cytometry; expression of innate **and adaptive** resistance factors within the foreskin and rectosigmoid mucosa by real-time PCR and antibody-based detection; antigen-specific T-cells within the foreskin and rectosigmoid mucosa evaluated by ICS

B Revised in Section 9.4

9.4 Immunological assays

9.4.1 Antibody-based detection

Cytokines, **immunoglobulins,** and chemokines will be evaluated in plasma**, mucosal sample lysates,** and supernatants of explant cultures with the Luminex multiplex assay and/or enzyme-linked immunosorbent assay (ELISA). Markers of immune activation in HIV target cells will be examined in plasma specimens from weeks 2, 3, 4, 5, 10, 26, 27, and 28 and will be compared to baseline samples collected at weeks 0 and 1. For example, levels of CCR5 and CXCR4 binding chemokines, such as RANTES, MIP1α, MIP1β, and SDF-1 may be examined in plasma and explant culture supernatants. **Human serum albumin may be**

measured as a control. Data will be reported as concentration of these markers at said timepoints.

9.4.2 Real time (RT)-PCR

RNA isolated from PBMCs, foreskin tissue, rectal biopsies, and sigmoid biopsies will be used to measure bulk levels of chemokine receptors CCR5 and CXCR4, and their ligands RANTES, MIP1α, MIP1β, and SDF-1 *in situ*. Other markers of cellular activation (eg, Ki67, TNF-α)**, markers of immune cells,** and proteins promoting innate antiviral protection (eg, langerin, APOBEC3, and TRIM5α) may be analyzed.

…

9.4.4 Histopathology and immunofluorescence microscopy

Formalin-fixed and paraffin-embedded foreskin, sigmoid, and rectal tissue samples will be stained for immunofluorescence microscopy to analyze the keratin layer thickness and the localization of HIV target cells within the epidermis and dermis. Paraffin-embedded samples will also be used to characterize **B cells and** CD4+ T cells in tissue, which will be tested for correlation with the flow cytometry approach. CD4+ and CD8+ T cells will be measured in addition to CCR5 levels, Ki67 levels, and their proximity to the external surface of the mucosa.

Protocol modification history

Protocol modifications are made to HVTN protocols via clarification memos, letters of amendment, or full protocol amendments. HVTN protocols are modified and distributed according to the standard HVTN procedures as described in the HVTN Manual of Operations (MOP) (Organization and Policy>Vaccine Selection and Protocol Development).

The version history of, and modifications to, Protocol HVTN 914 are described below.

Date: February 8, 2012

Protocol version: 1.0
Protocol modification: Letter of Amendment 3

Item 1 Clarified in Section 5.2, *Secondary objectives and endpoints* Section 9.4, *Immunological assays*: Humoral immune activity in foreskin

Date: January 4, 2012

Protocol version: 1.0
Protocol modification: Letter of Amendment 2

Item 1 Revised in Sections 5.3, 8.6, 8.7, 9.4.5, and Appendices A, B, F, and G: HIV surveillance enhanced

Date: November 29, 2011

Protocol version: 1.0
Protocol modification: Letter of Amendment 1

Item 1 Clarified in Sections 3, 6.1, and 7.3: Sample size, participant withdrawal, replacement, and termination

Item 2 Clarified in Appendix A, *Sample informed consent form*: Enemas prior to flexible sigmoidoscopies may occur at home or in the clinic

Item 3 Clarified in Appendix A, *Sample informed consent form*: Samples shipped out of country for analysis

Item 4 Clarified in Appendix F, Laboratory procedures: Resolution of indeterminate HSV-2 test results

Date: December 8, 2010

Protocol version: 1.0
Protocol modification: Clarification Memo 2

Item 1 Clarified in Section 7.1, *Inclusion criteria*: INR criterion

Item 2 Clarified in Appendix F, *Laboratory procedures*: Processing and assay locations for NG/CT urine specimens

Item 3 Corrected in Appendix G, *Procedures at CRS*: Abbreviated physical exams at Weeks 0 and 2

Item 4 Updated in Protocol team list: Statistician, Clinical trials manager, and Project manager

Date: September 29, 2010

Protocol version: 1.0
Protocol modification: Clarification Memo 1

Item 1 Clarified in Section 9.4.2, *Real time (RT)-PCR* and Appendix F, *Laboratory procedures*: RT-PCR performed on whole blood collected in Tempus tubes

Date: August 16. 2010

Protocol version: 1.0
Protocol modification: NA

Original protocol
