# Supplementary material for: Transient Peripheral Immune Activation follows Elective Sigmoidoscopy or Circumcision in a Cohort Study of MSM at Risk of HIV Infection
Source: PLoS One. 2016 Aug 18;11(8):e0160487. doi: 10.1371/journal.pone.0160487 (PMC4990246; doi:10.1371/journal.pone.0160487)
Supplement: S1 Table — (DOCX) [file pone.0160487.s003.docx]

**Supplementary Table S1. Results from flow cytometry analysis comparing pre- and post-procedure expression of activation and migration markers in peripheral CD4+ T cells.***

|  | **Changes post-sigmoidoscopy** | | **Changes post-circumcision** | | |
| --- | --- | --- | --- | --- | --- |
| **CD4+ T-cell Phenotype** | **Previous to sigmoidoscopy (week 2)** | **1 week post sigmoidoscopy (week 3)** | **Previous to circumcision (week 4)** | **1 week post circumcision (week 5)** | **6 weeks post circumcision (week 10)** |
| **αE+β7+** | n=22  Median 0.4%  IQR 0.2-0.5 | n=21  Median 0.4%  IQR 0.3-0.5  p=0.6995 | n=19  Median 0.4%  IQR 0.3-0.5 | n=19  Median 0.4%  IQR 0.3-0.5  p=0.5459 | n=19  Median 0.4%  IQR 0.3-0.6  p=0.1688 |
| **CLA+** | n=22  Median 1.3%  IQR 0.8-2.5 | n=21  Median 1.7%  IQR 1.1-2.7  p=0.4684 | n=19  Median 1.8%  IQR 0.8-2.3 | n=19  Median 1.3%  IQR 0.8-1.9  p=0.0602 | n=19  Median 1.6%  IQR 0.7-2.4  p=0.6226 |
| **Ki67+ Bcl2 low** | n=22  Median 0.8%  IQR 0.6-1.2 | n=21  Median 1.0%  IQR 0.7-1.3  p=0.1337 | n=19  Median 0.7%  IQR 0.6-1.1 | n=19  Median 0.8%  IQR 0.7-0.9  p=0.7680 | n=19  Median 1.0%  IQR 0.7-1.3  p=0.0204 |

*p-values are Wilcoxon sign rank tests comparing paired results from day of the procedure and respective healing periods.
